# Emerging switchable ultraviolet photoluminescence in dehydrated Zn/Al layered double hydroxide nanoplatelets

**DOI:** 10.1038/s41598-019-48012-8

**Published:** 2019-08-08

**Authors:** G. Prestopino, G. Arrabito, A. Generosi, A. Mattoccia, B. Paci, G. Perez, G. Verona-Rinati, P. G. Medaglia

**Affiliations:** 1 Dipartimento di Ingegneria Industriale, Università di Roma ‘Tor Vergata’, Via del Politecnico 1, I-00133 Roma, Italy; 2 Dipartimento di Fisica e Chimica, Università degli Studi di Palermo, Ed.17, V.le delle Scienze, 90128 Palermo, Italy; 3 Istituto di Struttura della Materia, Consiglio Nazionale delle Ricerche (ISM-CNR), Area di Ricerca di Tor Vergata, Via del Fosso del Cavaliere, 100, 00133 Rome, Italy; 4 Istituto di Struttura della Materia, Consiglio Nazionale delle Ricerche (ISM-CNR), Via Salaria km 29.300, Roma, Monterotondo Scalo, 00015 Italy

**Keywords:** Two-dimensional materials, Two-dimensional materials

## Abstract

Layered double hydroxides show intriguing physical and chemical properties arising by their intrinsic self-assembled stacking of molecular-thick 2D nanosheets, enhanced active surface area, hosting of guest species by intercalation and anion exchanging capabilities. Here, we report on the unprecedented emerging intense ultraviolet photoluminescence in Zn/Al layered double hydroxide high-aspect-ratio nanoplatelets, which we discovered to be fully activated by drying under vacuum condition and thermal desorption as well. Photoluminescence and its quenching were reproducibly switched by a dehydration–hydration process. Photoluminescence properties were comprehensively evaluated, such as temperature dependence of photoluminescence features and lifetime measurements. The role of 2D morphology and arrangement of hydroxide layers was demonstrated by evaluating the photoluminescence before and after exfoliation of a bulk phase synthetized by a coprecipitation method.

## Introduction

**Figure 1:**
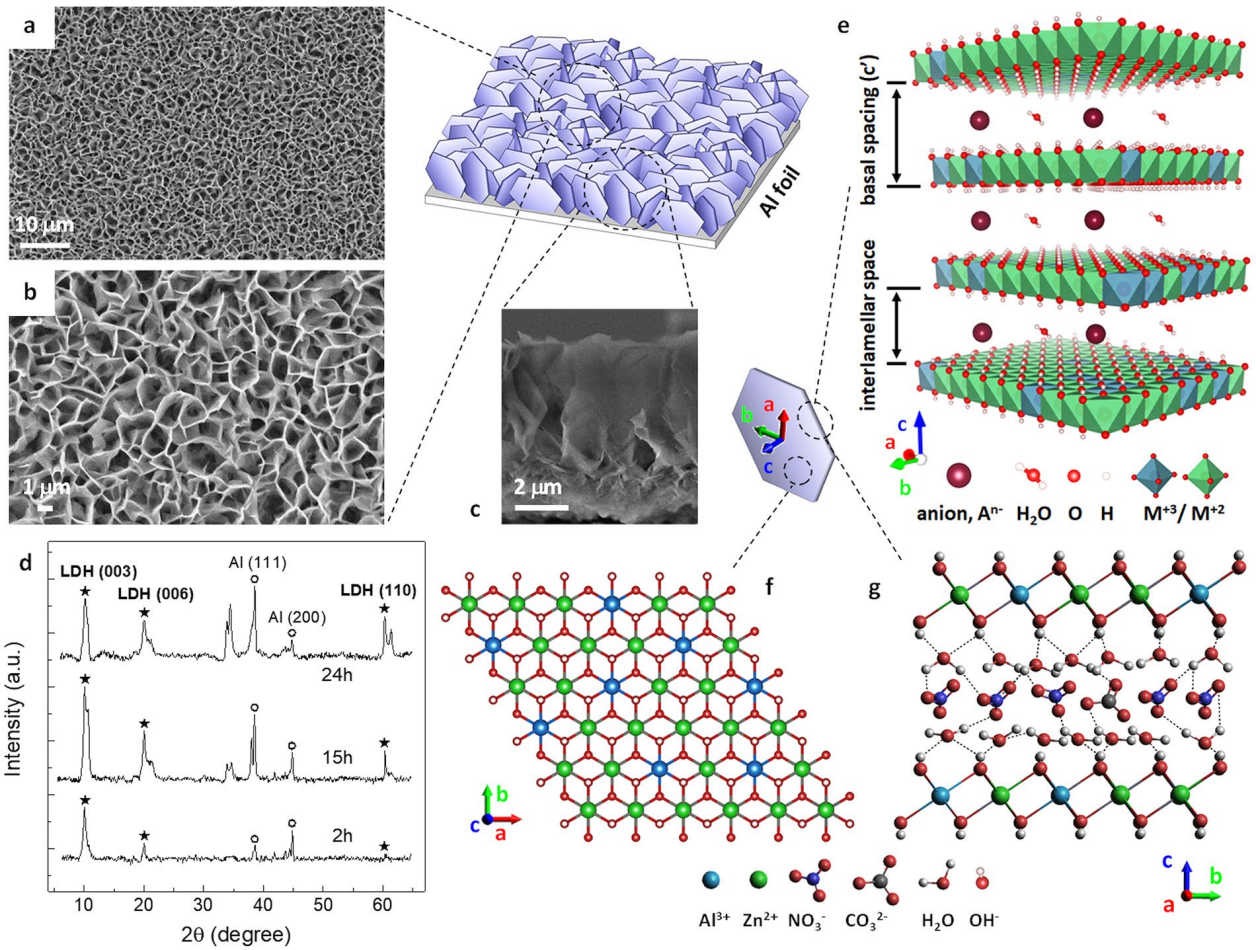
Schematic three dimensional (3D) illustration of the Zn/Al LDH film grown on Al foil substrate. (**a**,**b**) Scanning electron microscopy (SEM) images at two different magnifications showing the top surface morphology of the Zn/Al LDH film. (**c**) Cross-sectional SEM image of the film. (**d**) XRD patterns of the as-prepared Zn/Al LDH films on Al foils for three different growth times (2 h, 15 h, 24 h). (**e**–**g**) Structural modelling relevant to the single LDH crystallite. (**e**) Schematic 3D representation of the ${{\rm{M}}}_{1-x}^{2+}{{\rm{M}}}_{x}^{3+}{({\rm{OH}})}_{2}{{\rm{A}}}_{x/n}^{n-}\cdot m{{\rm{H}}}_{2}{\rm{O}}$ LDH general structure. (**f**) Top- and (**g**) side-views of two dimensional structure models of Zn/Al $({{\rm{NO}}}_{3}^{-})$-LDH layers along the c-axis and the a-axis, respectively. A simplified representation of the complex hydrogen bond network among hydroxyl groups, water molecules, and anions is sketched.

Two dimensional (2D) materials have drawn enormous interest in the recent decades since they present unique physical, optical and chemical properties that are distinct from or absent in their bulk counterparts, arising from their ultimate in-plane anisotropy and confinement effects^1–5^. In particular, their nanophotonic properties and applications^4^ and intriguing photoluminescence (PL) properties^2^ have attracted an increasingly burgeoning research. Layered crystals, exhibiting strong in-plane covalent bonding and weak out-of-plane van der Waals, electrostatic or hydrogen bonding, are the typical bulk precursors of 2D materials. Layered metal oxides^6^ and layered double hydroxide (LDH) materials^7^ are a class of inorganic lamellar compounds consisting of alternately stacked charged polyhedral layers accompanied by charge-balancing ions^8^. The 2D elementary building blocks of these layered structures are charge-bearing inorganic macromolecule-like monolayers, typically obtained by liquid exfoliation of their parent layered bulk precursors^8,9^. LDHs, also known as hydrotalcite clays, consist of alternately stacked positively charged brucite-like host layers and weakly bound hydrated interlayers containing intercalated charge-balancing anions and water molecules. LDHs (see the schematics in Fig. 1) can be described as ${{\rm{M}}}_{1-x}^{2+}{{\rm{M}}}_{x}^{3+}{({\rm{OH}})}_{2}{{\rm{A}}}_{x/n}^{n-}\cdot m{{\rm{H}}}_{2}{\rm{O}}$, where M^2+^ (Mg, Zn, Cu, Ni etc.) and M^3+^ (Al, Cr, Co, Ga etc.) are di- and trivalent metal cations, respectively, *x* is the molar ratio of the trivalent cation [*x* = M^3+^/(M^2+^  + M^3+^)], which typically^10^ varies between 0.20 and 0.33, A^*n*−^ (e.g., ${{\rm{CO}}}_{3}^{2-}$, ${{\rm{NO}}}_{3}^{-}$, ${{\rm{ClO}}}_{4}^{-}$, ${{\rm{SO}}}_{4}^{2-}$) is an anion with charge *n*, and *m* is the amount of intercalated water^10,11^. The isomorphous substitution of a fraction of divalent cations with the trivalent ones generates positively charged layers consisting of edge-sharing octahedral M(OH)_6_ units. These layers vertically stack on top of each other and the resulting positive charge is compensated by intercalated anions. Water molecules fill the interlayer space and are connected to both the metal hydroxide layers and the interlayer anions forming an extensive hydrogen bond network^10,12–14^. LDHs have found applications such as catalysis^15,16^, photocatalysis^5,17,18^, drug release^19^, energy conversion and storage^20^. Exfoliation of bulk LDHs^15,16,20–23^ into single layer nanosheets has been shown to greatly enhance electrocatalytic and photocatalytic activity^15–18^, and hydroxyl ion conductivity^24^. Thickness reduction of bulk layered LDHs to few-layer thick nanosheets, up to the monolayer limit, enhances 2D anisotropy, electrochemical surface area (ECSA) and number of active edge sites^15–18^. In this respect, some intriguing analogies in catalysis mechanisms and edge effects have been reported between LDH and 2D layered transition metal dichalcogenide (TMD) MoS_2_ nanosheets^15,16,25^. TMDs are one of the most popular 2D layered materials exhibiting effective catalysis activity^26^ and ultimate PL properties arising from indirect to direct bandgap transition as the monolayer limit is approached with exfoliation^27,28^. It is worth to point out that many similarities in structural and functional features can also be found between LDHs and layered two dimensional hybrid halide perovskites, where bulky interlayer organic spacer cations are sandwiched between corner-sharing inorganic MX_6_ metal (M) halide (X) octahedral layers, held together by weak van der Waals and intermolecular forces^29,30^. In particular, 2D Ruddlesden-Popper perovskites (RPPs) are natural self-assembled quantum-well (QW) like materials^31^, described by the generic formula (A′)_2_(MA)_*n*−1_M_*n*_X_3*n*+1_, where A′ is a long-chain organic spacer, MA is a small organic cation, and *n* is the number of perovskite MX_6_ monolayers per unit cell (e.g., *n* = 1 corresponds to a single octahedral thick LDH-like structure). Owing to their inherent QW structure, 2D-layered perovskites have strongly bound excitons with enhanced binding energies, manifesting in stronger, higher quantum yield and blue-shifted PL compared with that of their 3D counterparts.

To date, photoluminescence properties of LDH high-aspect-ratio particles, including LDH nanoplatelets in self assembled interconnected hierarchical structures, or nanosheets obtained by exfoliation procedure or delamination are still far from clear. Tuneable PL was reported for hybrid LDHs incorporating rare-earth luminescent complexes in the cationic host layers and/or intercalating photofunctional anions in the interlamellar spaces^32^. Despite that, photoluminescence of pristine LDHs has received less attention and only few studies have reported on PL from colloidal, powder-like, and thin film LDHs^33–37^. A broad structured emission band in the range of 350–550 nm is coherently reported, whose peaks are generally attributed to surface defects. Interestingly, two recent works on PL from Mg/Al and Zn/Al LDHs^36,37^ report an enhancement of PL emission in the UV region (360–400 nm) after annealing. In the work by Zhou *et al*.^37^, the PL emission of a nano film of Zn/Al LDHs grown on nano porous anodize alumina was measured at room temperature after annealing procedure (500 °C, 5 h), and two weak UV emission peaks at 380 nm and 396 nm were attributed to radiative inter-band transitions from conduction to valence band and to exciton recombination, respectively. The obvious potentialities of LDHs as a 2D direct bandgap semiconductor with unique undiscovered PL properties is therefore a still unexplored research area. The similarities to highly luminescent layered 2D materials, such as TMDs and 2D-layered perovskites are highly motivating, as well.

Here we report the emergence of an unprecedented intense UV photoluminescence in Zn/Al LDH high-aspect-ratio nanoplatelets which we found to be fully activated to different extents by dehydration in vacuum and by thermal desorption. Such PL, whose most intense features are two peaks centred at 375 nm and 396 nm, was completely absent in air, where only a broad blue band similar to that reported in previous studies^33–37^ could be measured. Dehydration – hydration resulted to be a completely reversible process that reproducibly switched the emerging intense PL and its quenching. Surprisingly, only LDH nanoplatelets exhibited such PL behavior. Bulk LDH powders did not show any luminescence in vacuum as long as measurements were repeated after exfoliation and drop casting on a silicon substrate as a support. Our results experimentally demonstrate, for the first time, unique and previously unknown PL properties of pristine Zn/Al-LDHs, showing also intriguing analogies with other layered 2D materials widely reported in the recent literature. In particular, analogy is twofold: the strong influence of adsorbate dynamics on the observed optical transitions in self-assembled LDH nanoplatelets, and the role of two-dimensionality in PL emission as high aspect-ratio clusters were produced by exfoliation of bulk LDH crystals.

## Results

Zn/Al-LDH nanoplatelets with ${{\rm{NO}}}_{3}^{-}$ [Zn/Al $({{\rm{NO}}}_{3}^{-})$-LDH] were grown onto commercial aluminum foils by an *in situ* one-step growth method already reported in our previous works^38,39^. The growth mechanisms and the effectiveness of this method in allowing synthesis of high aspect ratio few layered LDH nanoplatelets on metallic or metal-coated substrates, with controlled morphology, strong adhesion to the substrate, chemical stability, and high active surface area, are well documented^11,37,40–43^. Details of the growth technique for the prepared LDH samples are presented in the Methods section. Figure 1 shows a schematic three dimensional (3D) representation of the quasi-vertically aligned hexagonal nanoplates forming the Zn/Al-LDH film on the Al foil substrate. Scanning electron microscopy (SEM) images showing the top surface morphology are reported in Fig. 1a,b. Closely interconnected flexible nanoplatelets extending all over the Al substrate form a hierarchical self-assembled porous structure which is typical of Zn/Al-LDHs grown on metal substrates^11,38,40,41,43^. The LDH crystallites are almost randomly oriented, lying both nearly perpendicular and aslant with respect to the Al substrate. A cross-sectional SEM image of the Zn/Al-LDH film is shown in Fig. 1c. Morphology and crystallization process of Zn/Al-LDHs grown on Al substrates are comprehensively discussed in previous studies^11,42,43^. Some Zn/Al-LDH nanoplatelets were scraped from the Al foils and elemental composition analysis by means of energy dispersive X-ray spectroscopy (EDX) revealed an average Zn/Al ratio equal to about 2.0 for all the prepared LDH samples (a summary of EDX data is given in Supplementary Fig. S1). The X-ray diffraction (XRD) patterns confirmed the LDH structure of the prepared films, exhibiting fairly sharp (*00 l*) reflections typical of the lamellar stacking sequence of well-crystallised hydrotalcite-like materials. XRD spectra by three Zn/Al $({{\rm{NO}}}_{3}^{-})$-LDH films grown on Al foils for growth times equal to 2 h, 15 h, and 24 h are shown in Fig. 1d, where the basal (003) and (006) and the non-basal (110) reflections are indexed, as well as the (111) and (200) reflections by the Al substrate, which were used as a reference for the 2θ scale (a detailed indexing of the XRD spectrum is shown in Supplementary Fig. S2). The presence of both basal and non-basal reflections well agrees^11^ with the almost randomly oriented LDH nanoplatelets as shown in Fig. 1a,b. The measured diffraction peaks can be indexed to the hexagonal unit cell with R-3m rhombohedral symmetry commonly used for the description of the LDH structures^10,44^. The average unit cell parameters for the evaluated LDH samples were therefore calculated accordingly (for a complete summary see Supplementary Table S1). A basal spacing of 0.881 nm was calculated by the formula *c’* = 1/2·(*d*_003_ + 2*d*_006_), and an interlamellar space of 0.40 nm can be estimated assuming a 0.48 nm thick brucite-like sheet^10,32,44^ (see Fig. 1e for a schematic 3D representation of the LDH general structure). An average *c* axis parameter equal to 2.643 nm and a lattice parameter *a* = 0.306 nm were calculated by *c* = 3·*c’* and *a* = 2·*d*_110_. These values are consistent with those reported in literature for Zn/Al $({{\rm{NO}}}_{3}^{-})$-LDHs with an approximate molar ratio *x* = 0.33 (i.e. a 2:1 Zn/Al ratio)^10,11,45^, which, in turn, was verified experimentally for our prepared LDH films by means of the EDX analysis. This molar ratio was demonstrated^10,44,45^ to produce well defined LDHs with high crystallinity and no formation of other phases with low crystallinity (e.g., ZnO phase and ZnAl_2_O_4_ spinel). The presence of ZnO nanorods (NRs) in the fabricated LDH films was unlikely as well, due to the thick Al substrate of our LDH samples which acts as an infinite reservoir of Al in the nutrient solution^38^. Figure 1f,g show a schematic representation of top and side views, respectively, of the structure model for the Zn/Al $({{\rm{NO}}}_{3}^{-})$ hydroxide layers. Hydroxyl groups and water molecules abundantly populate outer and inner surfaces of the stacked macromolecule-like metal hydroxide layers as well as their edges and interlayer spaces, governing dynamics and physicochemical properties of nanostructured LDH crystals and leading to a vast range of temperature- and humidity-driven structural transitions^46,47^. Structural water molecules fill the interlayer spaces with a dense network of hydrogen bonds to the interlayer anionic guests and to the hydroxyl groups pointing toward the interlayer galleries.

The influence of hydration on structural and electronic properties of LDHs and the effect of dehydration have been extensively studied both experimentally and by means of density-functional theory (DFT) simulation^12–14,44,46–50^. Water content in the interlayer spaces influences basal spacing, exfoliation energy, orientation of anionic guests as well as charge transfer among water molecules, host layers, and anions, band structure and band-gap energy. Nothing is known, however, about possible PL activation in pristine LDHs by desorption of luminescence quenchers.

**Figure 2:**
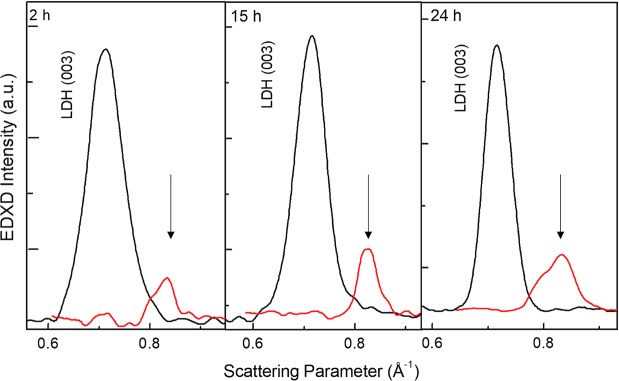
EDXD patterns. Low angle region comparison of pristine (black line) and annealed (red line) Zn/Al $({{\rm{NO}}}_{3}^{-})$-LDH films (growth times 2 h, 15 h, 24 h). *In-situ* measurement allows for an accurate determination of the basal plane shrinking, as evidenced by the arrows, associated to dehydration caused by thermal annealing.

To get better insight on the dehydration role on the LDH structure, energy dispersive X-ray diffraction (EDXD)^51^ was performed. The EDXD patterns were acquired *in-situ* on the LDH samples under thermal treatment. First, the deposited film crystallographic signature was accurately detected (together with the substrate signal) spanning the whole range of interest of the scattering parameter *q* (working at different scattering angles *ϑ*). The results (see Supplementary Fig. S3) are in agreement with the *ex-situ* XRD investigation results reported above. Subsequently, the geometry of the experimental set-up was kept fixed (at *ϑ* = 0.90°) and *in-situ* measurements where performed before and after a thermal annealing performed *in-situ* (10 minutes at 100 °C), thus ensuring that the same scattering volume was sampled during each acquisition. In this way, a direct comparison of the EDXD patterns was possible, providing a quantitative information on the crystallographic rearrangement occurring as a consequence of annealing. In Fig. 2, the basal (003) [Zn/Al $({{\rm{NO}}}_{3}^{-})$-LDH] reflection is shown for all pristine samples (black lines), matching an interplanar distance of 8.90 Å (*q* = 0.710 Å^−1^). After thermal treatment (red lines) dehydration occurred causing a contraction of the basal planes: the peak shifts to *q* = 0.830 Å^−1^, corresponding to a distance *d* = 7.50 Å, as evidenced by the arrows. A strong reduction of the crystalline signal was also observed. Interestingly, our result agrees very well with the reduction of the basal spacing from 8.9 Å to 7.2 Å reported for hydrated/ dehydrated [Li-Al-NO_3_] LDHs on heating at 50 °C in vacuum^46^.

**Figure 3:**
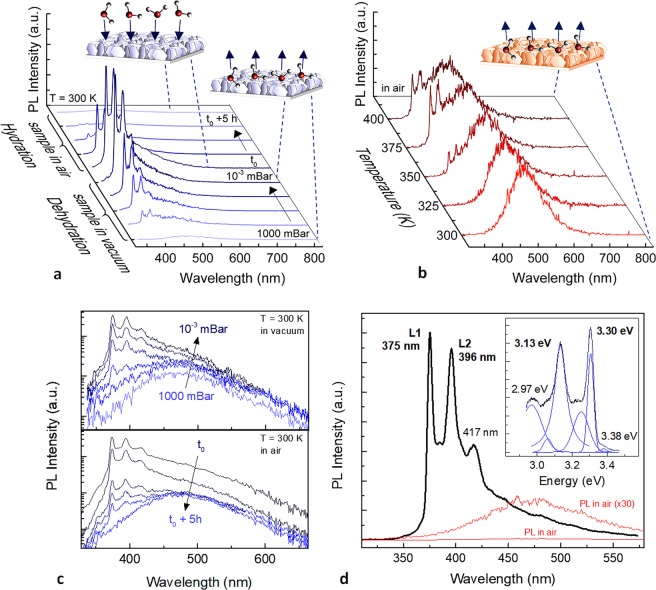
PL spectra of Zn/Al $({{\rm{NO}}}_{3}^{-})$-LDH nanoplatelets grown on Al foils showing the emergence of dehydration induced UV photoluminescence features and their reproducible quenching in the hydrated sample. (**a**) Room temperature PL spectra collected over a ∼5 minutes long dehydration process in vacuum (1000-10^−3^ mBar) followed by a 5 h re-hydration in ambient humid air, showing the reversible off-on-off process relevant to the emergence of the intense UV PL features. (**b**) PL spectra collected during a dehydration process induced by thermal annealing (300–400 K) in air. Sketches of desorption (dehydration) and adsorption (hydration) of adsorbates from/on LDH surfaces are also displayed. (**c**) PL spectra collected during the room temperature dehydration in vacuum and re-hydration in humid air reported in (**a**), plotted in a logarithmic scale. (**d**) Room temperature PL spectra collected in humid air at atmospheric pressure and in vacuum from respectively hydrated (red line) and dehydrated (black line) LDH films. In the inset a higher resolution spectrum (600 lines mm^−1^ grating) of the PL by a dehydrated LDH sample in vacuum is shown as a function of photon energy. Blue lines represent the individual Gauss-Lorentzian peaks and the resulting best fit of the experimental data.

The measured PL spectra from Zn/Al $({{\rm{NO}}}_{3}^{-})$-LDHs grown on Al foils as a function of hydration/ dehydration conditions are shown in Fig. 3. PL spectra collected over room temperature dehydration by outgassing in vacuum (1000-10^−3^ mBar) followed by a 5 h rehydration in humid ambient air, and those measured over dehydration by thermal annealing (300–400 K) are displayed in Fig. 3a,b, respectively. PL of pristine hydrated LDH nanostructures (stored and measured at 300 K in humid ambient air) consists of a nearly featureless broad PL band in the range 350–600 nm, which is consistent with previous studies^33–37^. Both dehydration procedures resulted in the emergence of sharp PL features in the UV region, with an almost complete suppression of the broad lower-energy emission in the case of RT outgassing in vacuum. Thermal annealing in the 300–400 K range, i.e. up to ∼130 °C, induces removal of physically adsorbed and interlayer water molecules, as demonstrated by thermogravimetric (TG) analysis reported in previous studies^32,35,40,45,49^. The increase in temperature, however, broadens and weakens the emerging UV PL features and the pristine broad PL feature is still well visible. The reversibility of the off-on-off switching relevant to the emergence of the intense UV PL features in the dehydrated sample and their quenching upon rehydration is shown in Fig. 3c, where PL signals of Fig. 3a are plotted in a logarithmic scale. Figure 3d shows the typical PL spectra collected at RT in humid ambient air and atmospheric pressure (red lines) and after outgassing in vacuum (black line). PL in vacuum is dominated by an intense PL feature at 375 nm accompanied by a vibronic progression. The two most intense UV PL features at 375 nm (3.30 eV) and 396 nm (3.13 eV) are denoted as L1 and L2, respectively. An energy spacing of about 170 meV (∼1371 cm^−1^) can be calculated between the L1 and L2 PL features. In addition, a low intensity and higher energy shoulder can be observed near 3.38 eV. The gap between this band and the highest energy emission feature L1 is about 80 meV (645 cm^−1^). The dehydration induced PL was well reproducible among all the fabricated LDH samples (see Supplementary Fig. S4), as well as the reversibility of the hydration induced quenching. In all cases, the switchable photoluminescence under dehydration in vacuum was found to be stable during several cycles. No photo-bleaching occurred during medium-/long-term reproducibility tests. Any contribution to the measured PL arising by possible residuals of chemicals mixed in the nutrient growth solution were excluded by numerical simulation of the solution chemical speciation (see Supplementary Fig. S5). More specifically, the simulation showed that, at the pH employed for the growth of LDHs (∼6), the nitrate anions do not form complex species with zinc ions, but are more favourably hosted in the interlamellar spaces of LDH given their negative charge. On the other hand, HMT mainly plays a role as pH regulator and slow supplier of OH^−^ ions. Given its lack of negative charges, it cannot be hosted in the interlamellar space. However, it cannot be ruled out that it could be adsorbed on the surface of LDH^52^ in the form of a dative covalent bond between the basic N donor atoms and Zn^2+^ ions which are a Lewis acid^53^. In this regard, Kumar *et al*.^52^ demonstrated by TG analysis that HMT can be desorbed from the surface of LDHs and that HMT contamination cannot be detected by EDX analysis on LDHs, likely because of the fact that under vacuum conditions HMT is desorbed from LDH. These observations clearly rule out any possible contamination issues deriving from the chemical solution bath employed for the synthesis of LDH. Additional tests were also carried out by measuring PL from pristine HMT salt samples, both in air and in vacuum, and a broad PL band in the visible was measured in both cases (see Supplementary Fig. S6).

**Figure 4:**
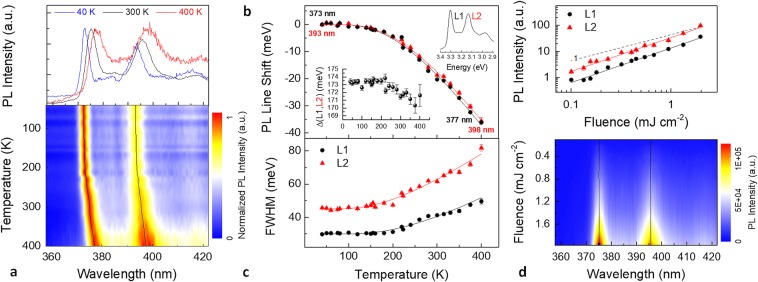
(**a**) Normalized PL of Zn/Al $({{\rm{NO}}}_{3}^{-})$-LDH nanoplatelets versus temperature. PL spectra at 40, 300, and 400 K and colour plot of the PL spectra measured at temperatures between 30 and 400 K. (**b**) Emission red-shift and (**c**) PL peak width (FWHM) of the L1 and L2 lines with their respective fits (solid lines) and their energy spacing Δ (inset) as a function of temperature. (**d**) Room temperature integrated PL intensity of L1 and L2 peaks (the solid lines are fits to a power law) and colour plot of the measured PL spectra as a function of the laser excitation fluence. Solid lines in the two photoluminescence maps are the peak positions of the L1 and L2 lines extracted by the least-squares fitted measured spectra.

In order to shed light on the photophysical properties of the observed PL features, a temperature dependent measurement was conducted in the 30 to 400 K range. Figure 4a shows the PL of a Zn/Al $({{\rm{NO}}}_{3}^{-})$-LDH film as a function of temperature. PL spectra are normalized against their peak height to better illustrate the temperature evolution of PL lineshape and linewidth. A colour plot of the PL spectra at temperatures between 30 and 400 K, and a set of PL spectra measured at 40, 300, and 400 K (upper inset) are displayed. Peak positions and full widths at half maximum (FWHM) of L1 and L2 peaks were extracted by least-squares fitting procedures of the collected spectra over the whole evaluated temperature range. The two solid lines in the PL map are the centres of the L1 and L2 lines. A non-normalized temperature-dependent PL map and the corresponding integrated intensities of L1 and L2 peaks as a function of temperature are shown in Supplementary Fig. S7. PL peaks exhibit a systematic red shift and a linewidth broadening as temperature increases. L1 and L2 peak positions as a function of temperature are well fitted using a standard semiconductor bandgap dependence^54^ of *E*_g_(*T*) = *E*_g_(0) − *S*〈*ℏω*〉[coth(〈*ℏω*〉/2*k*_B_*T)* − 1], where *E*_g_(0) is the ground-state transition energy at 0 K, *S* is a dimensionless coupling constant, and 〈*ℏω*〉 is an average phonon energy. The fitting parameters for L1 (L2) were *E*_g_(0) = 3.325 (3.152) ±0.001 eV, *S* = 1.5 (1.4) ±0.1, and 〈*ℏω*〉 = 61 (62) ±3 meV. Hence, it can be argued that the L1 and L2 emissions are related to an excitonic inter-band transition, rather than those associated with defect-related emissions^55^. Peak positions of the L1 and L2 lines and fitting curves (solid lines) are displayed versus temperature in Fig. 4b, subtracted by their respective *E*_g_(0) values. The energy spacing Δ(L1, L2) between the calculated centres of L1 and L2 slightly decreases as temperature increases, with an overall reduction of 3 meV between 30 and 400 K (Fig. 4b, inset). Figure 4c shows the FWHM data for the L1 and L2 emissions as a function of temperature. The measured temperature-dependent broadening of the peak FWHM Γ(T) is well fitted with the thermal broadening of the emission linewidth of most inorganic semiconductors due to exciton-phonon interaction, which can be expressed by the equation^56^ *Γ*(*T*) = *Γ*_0_ + *Γ*_ac_ + *Γ*_LO_ = *Γ*_0_ + *γ*_ac_*T* + *γ*_LO_/[exp(*E*_LO_/*k*_B_*T*) − 1]. Here, *Γ*_0_ is the temperature-independent inhomogeneous broadening term, which arises from scattering due to disorder and imperfections, and which corresponds to the linewidth at 0 K. The second and third terms (*Γ*_ac_ and *Γ*_LO_) are the homogeneous linewidth broadening which result from acoustic and longitudinal optical (LO) phonon (Fröhlich) scattering with exciton-phonon coupling strengths of *γ*_ac_ and *γ*_LO_, respectively, and the energy of LO phonon of *E*_LO_. The contribution due to acoustic phonons increases linearly with temperature. However, as it is clear by Fig. 4c, the gradient of the FWHM of L1 and L2 lines versus temperature approaches zero at low temperatures, suggesting a negligible acoustic phonon contribution (*γ*_ac_ ≈ 0). The linewidth broadening was therefore modelled fitting the experimental data using *Γ*(*T*) = *Γ*_0_ + *Γ*_LO_. In ref.^57^ a similar result for hybrid lead halide perovskites is ascribed to the dominant contribution of LO phonons over acoustic phonons in polar inorganic semiconductors. LDH is of course a highly polar material and our results are therefore clearly consistent with such model and well compare to those of other polar inorganic semiconductors^57^. The following fitting parameters were extracted for L1 (L2): *Γ*_0_ = 30.0 (45.0) ± 0.5 meV, *γ*_LO_ = 130 (120) ± 30 meV, and *E*_LO_ = 66 (53) ± 7 meV.

Figure 4d shows the room temperature dependence of PL intensity on the laser excitation density. In general^58^, the integrated PL intensity (*I*_L_) is proportional to *P*_exc_^k^, where *P*_exc_ is the excitation power density. *I*_L_ values relevant to the L1 and L2 peaks are displayed in a log-log plot as a function of the average laser fluence (mJ cm^−2^). The solid lines are the best fit to the data using a power-law dependence. A linear regression (*k* = 1, *I*_L_ ∼ *P*_exc_) is also displayed for comparison. For both peaks L1 and L2 an exponent factor of 1.3 was found over the whole evaluated excitation density range. This superlinear behavior (1 < *k* < 2) corresponds to a mixed bimolecular (*k* = 2) and monomolecular (*k* = 1) recombination of photoexcited carriers. Recently^31^, a nonlinear variation of PL with k ≈ 1.4 in exfoliated layered 2D perovskite crystals was ascribed to a partial dissociation of excitons to free carrier-like entities due to conversion/ dissociation of excitonic states to states associated with the layer edges. Indeed, edges, where lack of coordination occurs, are highly active sites^15–17,40,59^ in LDH nanosheets building blocks (including monolayers and few-stacked layers) and it is reasonable that they may influence the overall PL properties of LDH nanoplatelets as well. It is worth to point out that neither a line shift (see the solid lines in the colour plot) nor a change in the linewidth were observed in the measured spectra as a function of excitation density. The same measurement was repeated at the same irradiation condition on a second sample. In that case a superlinear behavior (*k* = 1.5) was measured up to a fluence of ∼0.1 mJ cm^−2^, followed by a sublinear dependence at higher fluence (*k* = 0.7). These results are reported in Supplementary Fig. S8.

**Figure 5:**
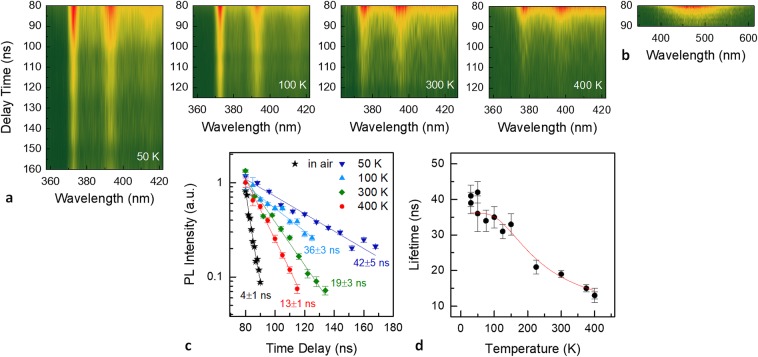
Spectrograms of time-resolved PL from a Zn/Al $({{\rm{NO}}}_{3}^{-})$-LDH sample measured (**a**) in vacuum at temperatures 50, 100, 300, and 400 K, and (**b**) in air at room temperature (i.e. pristine PL of the hydrated sample). (**c**) Semilogarithmic decay traces of the integrated PL intensities relevant to the L1 feature measured at 50, 100, 300, and 400 K. The PL intensity decay of the broad PL band from the hydrated sample is also displayed (black symbols). The solid lines are the best fit curves of the experimental data by a monoexponential decay function. PL integrated intensities were scaled to 1 for a better visual comparison. (**d**) Calculated lifetimes of L1 as a function of temperature and best fit curve (solid line).

Photogenerated charge-carrier recombination dynamics in our Zn/Al $({{\rm{NO}}}_{3}^{-})$-LDHs were investigated by temperature-dependent time resolved PL (TRPL) measurements. The PL decay from both vacuum-dehydrated and pristine hydrated samples was evaluated by measuring the time-gated PL emission with respect to the pulsed excitation. Figure 5a,b show the colour plots of TRPL spectra measured in vacuum (at temperatures of 50, 100, 300, and 400 K) and in air (at room temperature), respectively. The corresponding integrated PL intensities relevant to the L1 peak of the vacuum dehydrated sample and to the broad PL band of the hydrated sample are reported in the semilogarithmic decay plot displayed in Fig. 5c. PL dynamics exhibit a nearly mono exponential decay profile and the PL decay data were well fitted by a single exponential function (solid lines in Fig. 5c). Emission lifetimes ranged between ∼13 ns (400 K) and ∼42 ns (50 K) for the vacuum dehydrated LDH samples, whereas a markedly faster decay time of ∼4 ns (room temperature), near to detection limit, was found for the broad PL band of the hydrated sample. Figure 5d displays the lifetimes calculated for the L1 peak as a function of temperature. A shortening of the PL emission lifetime as temperature increases clearly indicates the onset of nonradiative transitions which dominate over radiative recombination. Indeed, the PL decay time *τ* can be modelled as a superimposition of a radiative recombination rate and an activated nonradiative decay^60^: 1/*τ* = *γ*_r_ + *γ*_nr_ exp(−Δ*E*/*k*_B_*T*), where *γ*_r_ = *τ*_r_^−1^ and *γ*_nr_* = τ*_nr_^−1^ are the inverse of radiative and nonradiative lifetimes, respectively, and Δ*E* the activation energy of thermal quenching. The solid line in Fig. 5d is the fit of the measured lifetimes according to this model, calculated with *τ*_r_ = 36 ± 1 ns, *τ*_nr_ = 6 ± 2 ns, Δ*E* = 45 ± 8 meV. The calculated emission lifetimes for the L2 PL feature and their variation with temperature were almost identical, within experimental error, to those of L1, therefore showing the same TRPL behavior (see Supplementary Fig. S9). The temperature dependence of the measured lifetime quenching is consistent with the FWHM broadening reported in Fig. 4(c) and may be reasonably related to nonradiative coupling via scattering with phonons.

**Figure 6:**
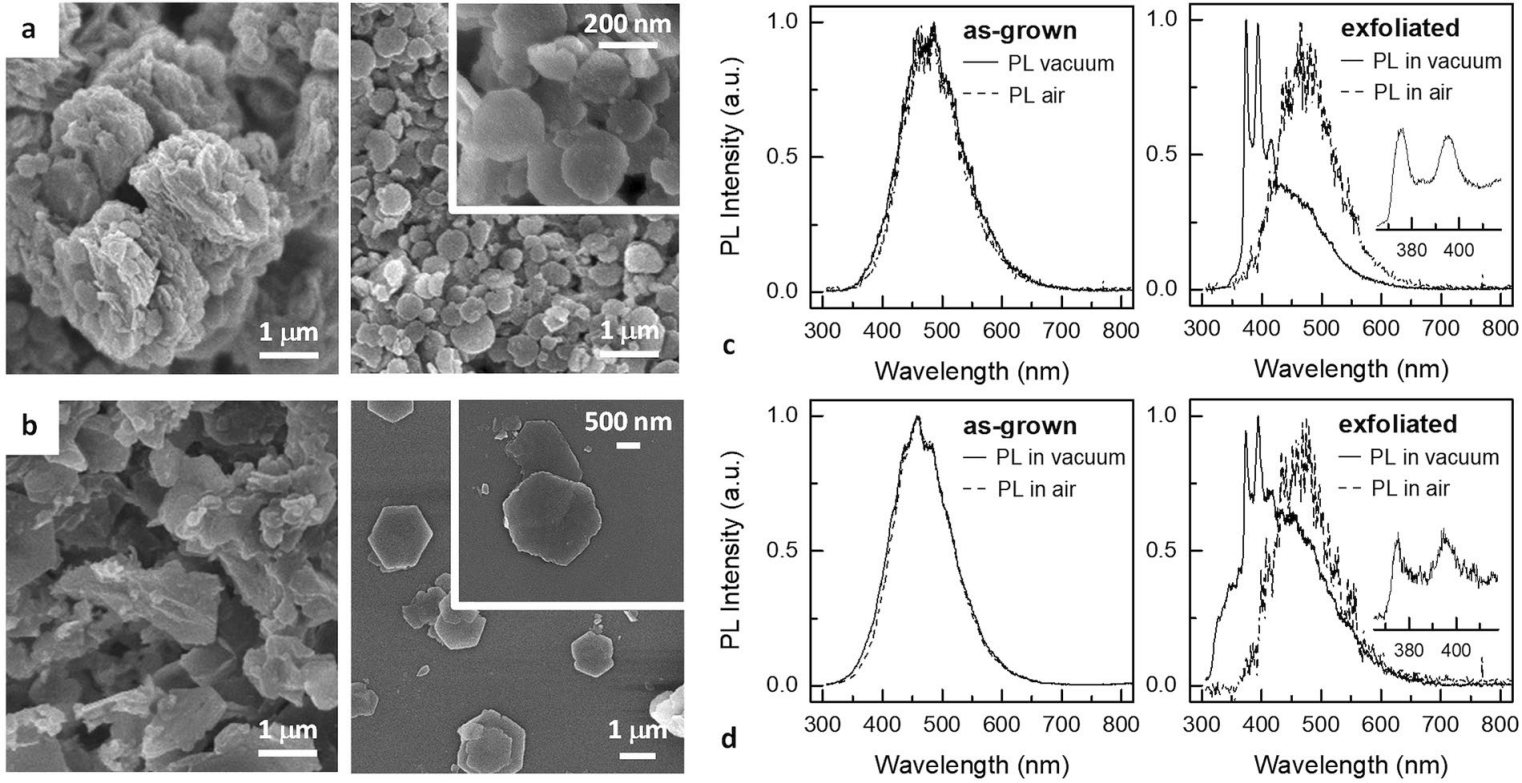
Exfoliation induced PL from (**a**,**c**) Zn/Al $({{\rm{NO}}}_{3}^{-})$, and (**b**,**d**) Zn/Al(Cl^−^) LDH samples in a bulk phase synthetized by a coprecipitation method. **(a**,**b**) SEM micrographs of the LDH samples: as-grown (left panels) and drop-casted on a SiO_2_/Si substrate after the exfoliation procedure (right panels). (**c**,**d**) Room temperature PL measured in air (dashed lines) and in vacuum (solid lines) from the as-grown LDH samples (left panels) and from the drop-casted nanoplatelets after exfoliation (right panels). Higher resolution spectra (600 lines mm^−1^ grating) of the UV emission are displayed in the insets.

The role of 2D anisotropy on the emergence of the intense narrow UV PL features from dehydrated LDHs was experimentally evaluated by means of a ‘top-down’ approach. Two types of bulk Zn/Al-LDH samples synthetized by a coprecipitation method, which differed in the intercalated anionic species (${{\rm{NO}}}_{3}^{-}$ and Cl^−^), were exfoliated by sonication in aqueous solution. EDX analysis revealed an average Zn/Al ratio equal to about 2.2 and 1.5 for the ${{\rm{NO}}}_{3}^{-}$ and Cl^−^ containing LDH bulk precursors, respectively (see Supplementary Fig. S1). LDH delaminated flakes were then drop-casted on SiO_2_/Si substrates (see Methods). Figure 6a,b show SEM images of the Zn/Al-LDHs before (i.e., as-grown powders) and after the exfoliation procedure. LDH aggregate morphology markedly changed after exfoliation, achieving a stacking of individual hexagonal nanoplatelets preferentially lying with their *ab*-planes parallel to the substrate. Figure 6c,d display the PL spectra measured in air (red dashed lines) and in vacuum (black solid lines) from the bulk and exfoliated LDHs. PL measured in air exhibited in all cases the broad band feature of the hydrated samples. Although the degree of dehydration was presumably the same for both samples, the intense narrow UV PL features surprisingly emerged from the exfoliated LDHs only, whereas PL from bulk samples was almost identical to that measured in air. As SEM images clearly show, exfoliation did not produce an increase in the overall exposed surface area, which was even reduced. The emergence of PL features after exfoliation must be therefore ascribed to an increase in density of PL-active sites. Recent studies have demonstrated that edges^15,16^ and surface atoms^5^ created by exfoliation of 2D layered materials, including MoS_2_ nanocrystals^26,28^, contain open coordination sites that might be the active sites for an enhanced catalytic activity. In particular, enhanced catalytic properties by comparing, in a top-down approach, oxygen evolution reaction (OER) activity of bulk LDHs, several-layer thick LDH nanoplatelets, and exfoliated LDHs were recently reported by Song and Hu^16^ and Liang and *et al*.^15^ The latter also suggested a change in the electronic structure of exfoliated LDH due to the changing interactions between the LDH layers and intercalants before and after exfoliation^15^. Our results seem to support the hypothesis that a similar mechanism may play a role in the emerging PL, as well as that the dehydration-induced PL in LDH nanoplatelets may possibly originate at the same active sites which lead to catalytic activity in LDHs.

## Discussion

Further combined experimental studies, DFT calculations, and theoretical analysis will be required to elucidate the precise mechanisms for the switchable PL measured in the LDH nanoplatelets as well as to rigorously identify PL active sites and exciton dynamics and yield insights into the relevant photophysics. For LDHs there are no available PL data in literature on such specific aspects for comparison purposes. However, analogies with other 2D materials and molecular-like systems, and some literature data from DFT simulations of LDHs, which also include hydrated and dehydrated Zn/Al $({{\rm{NO}}}_{3}^{-})$-LDHs, may help to briefly discuss our experimental results. DFT calculations for many M^2+^, M^3+^, *x*, and A^*n-*^ combinations relevant to the generic structure ${{\rm{M}}}_{1-x}^{2+}{{\rm{M}}}_{x}^{3+}{({\rm{OH}})}_{2}{{\rm{A}}}_{x/n}^{n-}$ demonstrate that LDH is a direct bandgap semiconductor, which is consistent with experimental results by UV-vis absorption and diffuse reflectance spectra. Bandgap values calculated by DFT for Zn/Al-LDHs range between 3.2 and 3.5 eV. A summary of some literature data is reported in Supplementary Table S2. Preliminary UV-vis absorbance measurements of Zn/Al $({{\rm{NO}}}_{3}^{-})$-LDH films were performed at room temperature and ambient condition to estimate bandgap energy. The results (see Supplementary Fig. S10 and Table S3) well agree with the literature data discussed above and in Supplemetarry Information. As a general result, the electronic properties of LDHs are mainly determined by the host matrices. The O-2p orbital is a main component of the Valence Band Maximum (VBM), and Conduction Band Minimum (CBM) is mainly composed of metal cation orbitals. As for Zn/Al-LDHs, a direct transition occurs between filled O-2p orbitals and unoccupied Zn-4s orbitals, with Al not making any obvious contribution to the electronic structure^18,48^. The band structure composition is therefore almost identical to that of ZnO^61^. Xu *et al*.^48^ noted that due to O-2p contribution to VBM, photogenerated hole tends to be localized in the O atom of the hydroxyl group, facilitating the oxidation of water molecules which strongly interacts with hydroxyl group via hydrogen bonding. This may support the hypothesis that in hydrated LDH samples PL quenching upon UV irradiation may be due to nonradiative transitions related to a photocatalytic activity. Moreover, Costa *et al*. showed that the extensive hydrogen bonding network by interlayer water molecules favours charge transfer between intercalated anions and hydroxyl slabs^13,14^, which may result in a path of non-radiative recombination as well. DFT simulation and XRD data available in literature, further supported by our EDXD results upon annealing, also provide information on structural change due to removal of interlayer water^46,47^. Dehydration determines a shrinking of interlayer galleries leading to a reorganization of intercalated anions which, for ${{\rm{NO}}}_{3}^{-}$ intercalated LDHs, is believed to be accompanied by a reorientation of the nitrate ion with its molecular plane lying parallel to the metal hydroxide layer^14,46^. DFT modelling based on our findings will be addressed to exactly correlate structural effects of dehydration in LDH nanosheets to changes in band structure and emerging active sites for PL, as well as to gain more insight into contributions of brucite-like hydroxyl layers and those of anionic interlayers.

Dehydration and exposure to ambient air reproducibly switched the emerging narrow intense UV PL features and the broad PL visible band, indicating a strong coupling of LDH photophysics to adsorbate dynamics, which influence surface, edge, and interlayer electronic and chemical states. Measurements of PL in controlled gaseous atmospheres will elucidate the role of specific adsorbates in quenching mechanisms. As demonstrated in Fig. 6, sample dehydration was necessary but was not a sufficient condition since the emergence of UV PL features was achieved for 2D high aspect-ratio LDHs only, clearly indicating the intrinsic 2D nature of the emerging exciton luminescence. This unprecedented behavior is in many ways similar to that of layered 2D TMDs^27,28^ and layered 2D perovskites (RPPs)^29,31^ in so far as the unique PL properties which emerge approaching the monolayer limit are concerned. These properties are determined by direct bandgap transitions and formation of tightly bound exciton states upon 2D confinement, with exciton binding energies of several hundred meVs, making them stable well beyond room temperature. For RPPs, their inherent QW structure, exhibiting a combination of quantum and dielectric confinement, further contributes to the formation of strongly bound excitons. Interestingly, Chen *et al*.^55^ reported enhanced exciton binding energy for ZnO nanosheets (100 nm thick uniform multilayer sheets), with intense UV emission and negligible defect-related visible emission, and propose the formation of a high-quality single-layered QW structure (i.e., air-ZnO nanosheet-air) as a possible contribution to exciton binding energy enhancement. A microcavity effect is also proposed to determine a positive optical feedback between the smooth nanosheet surfaces. Surface flatness and self-assembled QW-like structure of our LDH nanoplatelets may, in principle, support both these two exciton-light-coupling mechanisms. Electronic confinement in the brucite-like 4.8 Å thick layers of LDHs reasonably also contributes to induce stable excitons with high binging energy, which results in large oscillator strength and intense PL at room temperature. The emergence of the narrow intense UV PL features with a well-resolved vibronic structure, whose integrated intensity showed a negligible temperature dependence, is consistent with these effects.

The measured PL behavior vs. temperature (i.e., linewidth broadening, red-shift, and shortening of emission lifetime) exhibit strong exciton-phonon coupling. LDHs have obvious features of a highly polar ionic molecular crystal and strong Coulomb interaction must occur between excitons and lattice ions, enhancing electron-vibration coupling and reasonably resulting in the formation of electron and hole polarons. Polaron formation was demonstrated for 2D perovskites^62^ and low dimensional TMDs,^63^ and for ZnO as well^64^. Zn/Al-LDH band structure^48^ exhibit a dispersive conduction band and flat bands near the VBM which favour the formation of hole polarons. Similarly to 2D perovskites^62^, exciton confinement in the molecular thick LDH host layer may support small polarons, with formation of localized charge states which may couple to local structural lattice distortions. The measured well-resolved vibronic progression in the PL spectra, whose L1 and L2 features are the most intense emission lines, gives indication of an interplay of the localized exciton with lattice vibrations. Interestingly, the ∼170 meV vibronic spacing may be consistent with energies relevant to stretching modes of ${{\rm{NO}}}_{3}^{-}$ and ${{\rm{CO}}}_{3}^{2-}$ anions in the LDH interlayer (1380–1415 cm^−1^)^13,14,36,45,49^. A vibronic structure in the room temperature PL of a layered perovskite was recently reported^65^ and strongly localized electron-hole pairs were compared to excitons in organic chromophores, proposing the occurrence of Frenkel (localized) excitons rather than that of Wannier (delocalized) excitons, which are typical of semiconductors. Indeed, confinements effects in low-dimensional clusters lead to a change of electronic band structure to discrete molecule-like electronic states and PL features are similar to those of isolated molecules or complex ions.

## Conclusion

In conclusion, we have reported for the first time unprecedented PL properties of LDH nanoplatelets. Switchable PL from Zn/Al-LDHs was reproducibly tuned by dehydration and rehydration of LDH nanoplatelets, with an emerging intense UV PL and its quenching with a broad almost featureless visible emission band. The UV PL features were experimentally demonstrated to be strongly related to two-dimensionality and related photophysics. DFT results by previous reports and many similarities with other two dimensional semiconductors, in particular 2D layered perovskites, helped interpretation of our findings. Our work may open new insights into intriguing unexplored fundamental physics of pristine nanostructured LDHs and possible new applications as photofunctional materials responding to envinromental chemical or physical stimuli.

## Methods

### Synthesis of Zn/Al LDHs

Interconnected high-aspect-ratio Zn/Al $({{\rm{NO}}}_{3}^{-})$-LDH nanoplatelets on Al substrate were fabricated by reaction of a thin aluminum foil in hot aqueous solution of equimolar amounts of zinc nitrate hexahydrate [Zn(NO_3_)_2_·6H_2_O] and hexamethylenetetramine (HMT, C_6_H_12_N_4_) dissolved in deionized water. The Al foil acted as both the Al^3+^ source and the substrate supporting the interconnected LDH flakes. Commercially available 20 μm thick Al foils were pretreated in deionized water and ethanol and used as LDH growth substrate. Plain microscope slides 1 mm thick and 76 mm × 26 mm in dimension were used as a support, wrapped by the Al foils, and arranged, tilted by 45°, into a glass container, previously rinsed with hydrochloric acid and deionized water. The growth solution was prepared by dissolving 10 mM zinc nitrate hexahydrate [Zn(NO_3_)_2_·6H_2_O] (≥ 99.0%, Sigma-Aldrich) and 10 mM hexamethylenetetramine (C_6_H_12_N_4_) (≥ 99.0%, Sigma-Aldrich) in 250 mL deionized water. Hexamethylenetetramine was used as a pH regulator to control the solution basicity through the hydrolization and release of ammonia at high temperature. The growth was carried out in a preheated oven at 80 °C. Once desired growth time was completed, which was ranged between 2 h to 24 h, the sample was cooled down in ambient atmosphere, washed with ethanol and deionized water to remove residuals on the top of the LDH surface, and finally dried at 80 °C for 1 h in hot air oven. After that, the whitened LDH-covered Al foil was unwrapped from the glass slide and small samples, 1 cm × 1 cm typical size, were cut out for further tests. The same growth procedure was used for fabricating a Zn/Al $({{\rm{NO}}}_{3}^{-})$-LDH control sample on a silicon substrate. In that case, thin film aluminum coatings, 50–300 nm in thickness, were thermally evaporated (99.99999% purity Al wire from Umicore, Germany) onto the Si surface and used as the reactive metallic substrate for LDH synthesis. Two Zn/Al-LDH samples in a bulk phase, differing in the type of the nominally intercalated anions, were synthetized by a coprecipitation method. Zn/Al(Cl^−^)-LDHs were synthetized in an aqueous growth solution prepared with 20 mM zinc chloride (ZnCl_2_) (≥ 98.0%, Sigma-Aldrich) and 10 mM aluminum chloride hexahydrate (AlCl_3_·6H_2_O) (≥ 99.0%, Sigma-Aldrich). A nutrient solution containing 20 mM zinc nitrate hexahydrate (Zn(NO_3_)_2_·6H_2_O (≥ 99.0%, Sigma-Aldrich) and 10 mM aluminum nitrate nonahydrate (Al(NO_3_)_3_·9H_2_O) was used for the synthesis of Zn/Al $({{\rm{NO}}}_{3}^{-})$-LDHs. All the starting chemicals were used without further purification. For both synthesis procedures the resulting slurry was heated for 24 h in a preheated oven at 80 °C. The white precipitate was washed with deionized water many times and dried in a hot air oven at 70 °C for two days. Fragments of the dried LDH powders were peeled from the glass support and sonicated for 2 h in ultrapure deionized water. A droplet of the resulting dilute colloidal LDH nanoplatelets suspension was drop-casted on a SiO_2_/Si substrate, followed by quickly swabbing the droplet away and drying the Si substrate at 60° for 4 hours. Although both the exfoliation and the subsequent drop-casting were not fully optimized procedures, the adopted methodology allowed to some extent exfoliation of LDH bulk powders, and thin LDH nanoplatelets aggregates, were dispersed on the SiO_2_/Si substrates.

### Structural characterization

XRD measurements were carried out on as-grown Zn/Al LDH films synthetized on the aluminum foils. The XRD patterns were obtained with a PW 1130/00/60 Philips diffractometer (working at 40 kV, 30 mA) using CuK_α_ radiation (λ = 1.5405 Å) and collected over a 2θ range of 5°–65°, with a step size angle of 0.06°. The X-ray diffraction lines by the aluminum substrate, given by the (111) and (200) reflections, were used as a reference to calibrate the 2θ scale. Scanning Electron Microscopy secondary electron images were taken in a field emission scanning electron microscope (FE-SEM, model LEO SUPRA 1250, Oberkochen, Germany); semi-quantitative analysis of the elemental composition of the LDH samples used in this study was carried out using the EDAX energy dispersive X-ray (EDX) spectrometer (EDAX, INCA Energy 300, Oxford Inc., Abingdon, UK) attached to the FE-SEM. Energy Dispersive X-ray Diffraction measurements were performed by means of an unconventional experimental apparatus, developed in-house, working with a polychromatic X-ray source (W-anode tube, E_max_ = 50 keV, I = 30 mA), and accomplishing the photon detection by means of a Solid state Ge-single crystal detector (ORTEC). This experimental Bragg-Brentano configuration allows static measurements to be performed: using a white x-ray beam (no monochromator being present) no angular movement is necessary and the whole scattering parameter range of interest is explored by means of the energy sensitive detector. This static experimental setup, assuring that the same sample portion is probed, is particularly suitable when high precision *in-situ* measurements are to be performed, allowing to follow structural modifications over time.

### PL measurements

A Nd:YAG 5 ns pulsed 210–2400 nm tuneable optical parametric oscillator laser (Opolette laser by Opotek) was used as the photoexcitation source, focused to a spot beam area of about 6 × 10^−4^ cm^2^. Excitation wavelength was tuned to 280 nm (4.43 eV) and repetition rate was set to 20 Hz. Pulse energy (fluence) was varied in the range 0.01-1 μJ (∼0.02–2 mJ cm^−2^), and an average energy (fluence) of 0.3 μJ (0.5 mJ cm^−2^) was used for most measurements. The optics for beam focalization and PL light recoil consisted of three off-axis parabolic mirrors. PL light was focused onto the entrance slit of a 0.3 m triple grating spectrograph equipped with 150, 600, and 1200 lines mm^−1^ gratings (SpectraPro 3001, Roper Scientific), and detected using a gated multichannel-plate CCD camera system (PI-MAX, Roper Scientific), which enabled acquisitions with a nominal time resolution as little as 2 ns. The samples were mounted in a cold-finger liquid helium cryostat (CS-202AE, Advanced Research Systems) which allowed temperature variations in the 25–450 K range. A schematic of the experimental apparatus is illustrated in Supplementary Scheme S1. PL spectra were collected both in air (300–400 K) and in ∼10^−3^ mBar vacuum (30–400 K). In the latter case, the cold finger was enclosed in a radiation shield/vacuum shroud system and excitation laser as well as PL light passed through a high purity quartz (SiO_2_) window. The signal from a pyroelectric power meter was also recorded by a 500 MHz digital storage oscilloscope (WaveSurfer 454, Teledyne LeCroy) and used for estimating laser beam fluctuations and average pulse energy during acquisition of PL spectra. Except for the measurement of dependence of PL intensity on laser pulse energy, each collected PL spectrum was divided by the amplitude of the power meter signal averaged over the accumulation time of the respective PL measurement.

## Supplementary Information

Supplementary Information

## Data Availability

The experimental datasets produced during the current study are available from the corresponding author on reasonable request.
