# Supplementary material for: Emerging switchable ultraviolet photoluminescence in dehydrated Zn/Al layered double hydroxide nanoplatelets
Source: Sci Rep. 2019 Aug 8;9:11498. doi: 10.1038/s41598-019-48012-8 (PMC6687718; doi:10.1038/s41598-019-48012-8)
Supplement: Supplementary file 1 — Supplementary Information [file 41598_2019_48012_MOESM1_ESM.pdf]

SUPPLEMENTARY INFORMATION

Emerging switchable ultraviolet photoluminescence in dehydrated Zn/Al layered double hydroxide nanoplatelets

G. Prestopino,[a] G. Arrabito,[b] A. Generosi,[c] A. Mattoccia,[a] B. Paci,[c] G. Perez,[c] G. Verona-Rinati,[a] & P.G. Medaglia[a,*]

[a] Dipartimento di Ingegneria Industriale, Università di Roma 'Tor Vergata,' Via del Politecnico 1, I-00133 Roma, Italy.
[b] Dipartimento di Fisica e Chimica, Università degli Studi di Palermo, V.le delle Scienze, 90128 Palermo, Italy.
[c] CNR-ISM, Area di Ricerca di Tor Vergata, Via del Fosso del Cavaliere, 100, 00133, Rome, Italy.
[c] CNR-ISM, Monterotondo Scalo, 00015 Italy.

[*] Correspondence to: medaglia@uniroma2.it

S0. EXPERIMENTAL APPARATUS

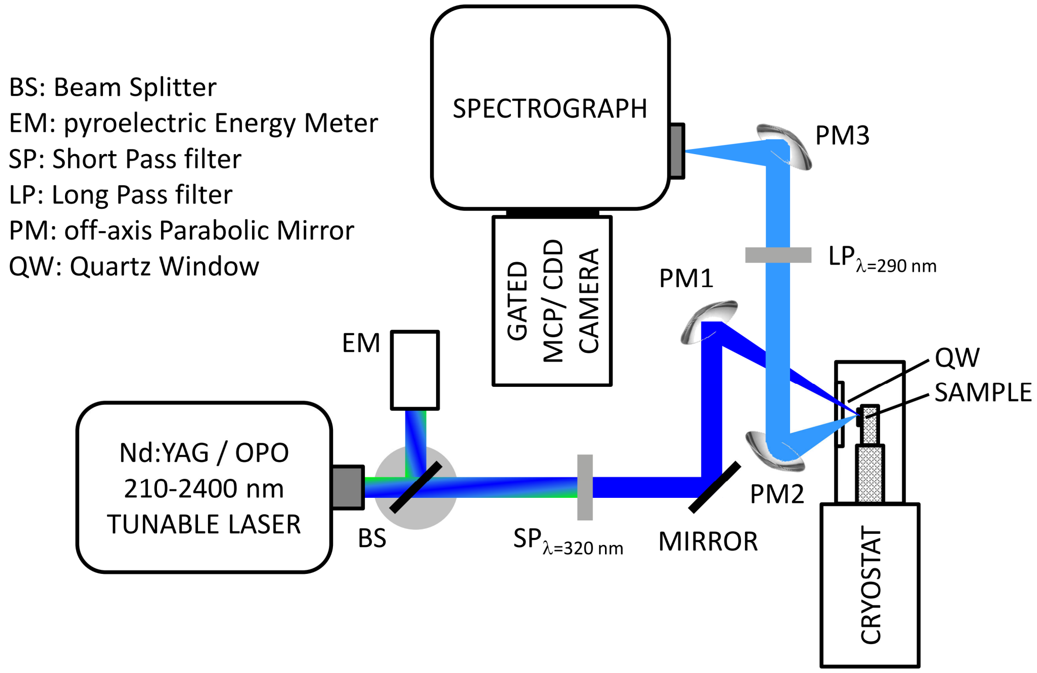

Scheme S1. Schematic representation of the PL apparatus used in the manuscript, with indication of the main components along laser photoexcitation beam and PL light paths. During all experiments the optical parametric oscillator Nd:YAG tunable laser was set at a 280 nm photoexcitation wavelength and 20 Hz pulse repetition rate. The cryostat system consisted of a double stage ARS CS-202AE cryostat (20–450 K) and a DE-202AE cold head shielded sample holder. It was mounted on a 3D moveable micrometric stage which enabled the positional control of the optical excitation beam spot over the sample. Three off-axis parabolic mirrors were used for beam focalization and PL light recoil. A short pass filter (λ = 320 nm) was placed downstream the beam splitter in order to block unwanted contamination of the photoexcitation light by the 560 nm wavelength laser line. A long pass filter (λ = 290 nm) was placed downstream the PM2 parabolic mirror in order to block any residuals of the 280 nm wavelength laser line which may contaminate PL light after being reflected on surfaces of sample and/or cryostat system.

S1. ENERGY DISPERSIVE X-RAY (EDX) ANALYSIS

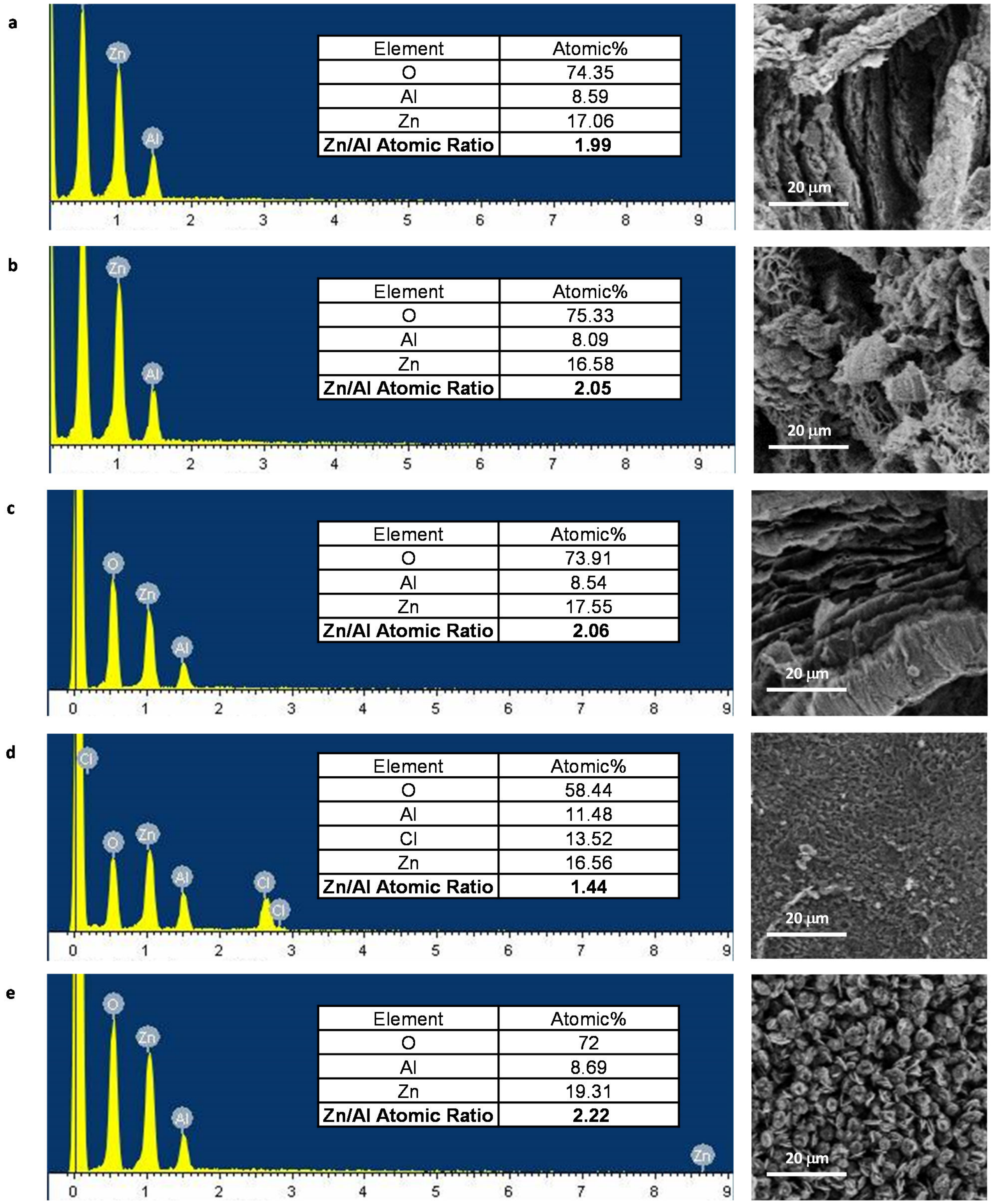

Figure S1. (Left panels) EDX data (Inca Suite software, version 4.13,Oxford Instruments, UK) and calculated Zn/Al atomic ratio for (a), (b), and (c) Zn/Al LDH samples scraped from aluminium foils supporting Zn/Al(NO_3^-)-LDH films whose growth times were 2 h, 4 h, and 24 h, respectively; (d) and (e) Zn/Al(Cl^-)-LDH and Zn/Al(NO_3^-)-LDH samples, respectively, synthetized by a coprecipitation method. (Right panels) Low magnification SEM micrographs of the respective LDH samples. For both EDX analysis and imaging the FE-SEM was operated with 5 keV accelerating voltage.

S2. XRD PATTERNS OF Zn/Al(NO_3^-)-LDH FILMS

X-ray diffraction (XRD) patterns of as-synthetized Zn/Al(NO_3^-)-LDH samples for growth times 2 h, 15 h, and 24 h are displayed in Figure S2 (a), (b), and (c), respectively. Experimental data are compared to XRD patterns calculated by VESTA software[1] (http://jp-minerals.org/vesta/en/) using cif files 3000048 and 9011602 from Crystallography Open Database (COD) as reference spectra for Zn/Al(NO_3^-)-LDH and aluminum, respectively (see Figure S2(d)).

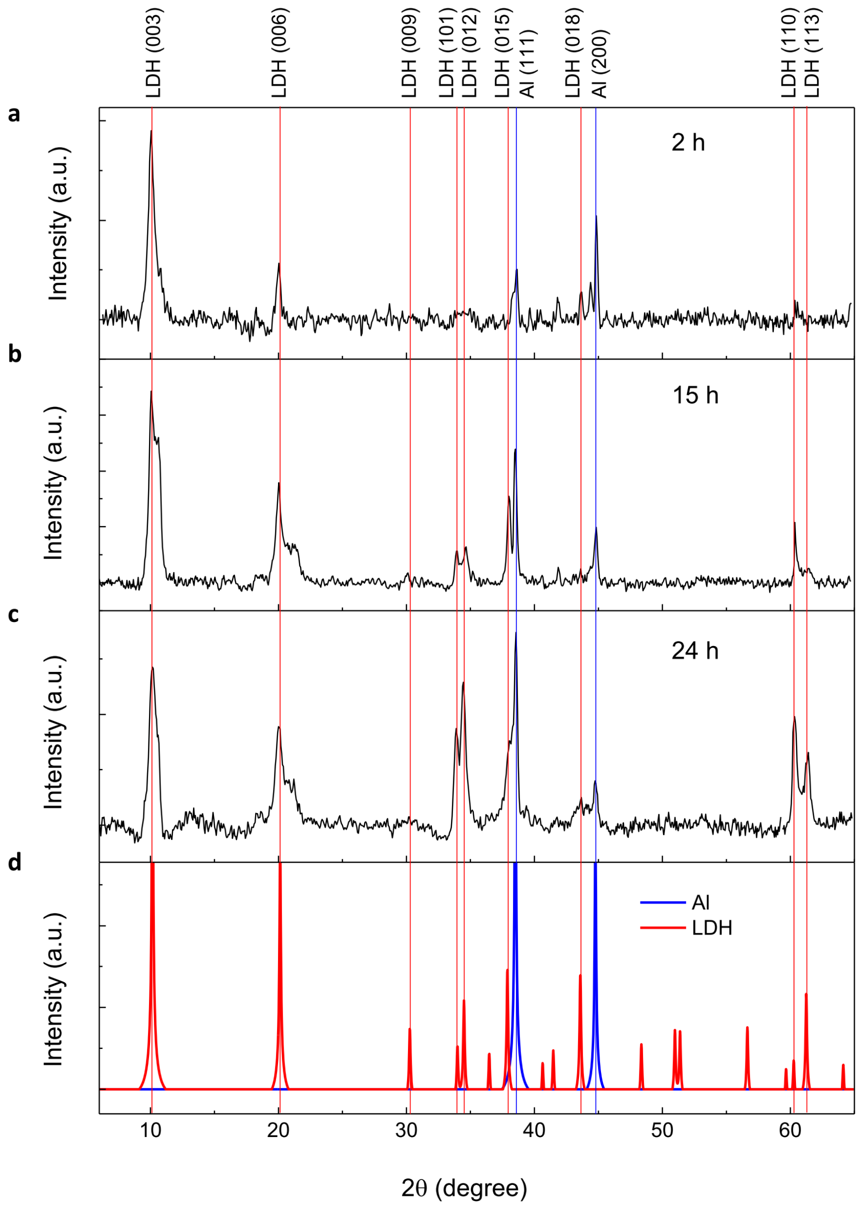

Figure S2. (a) – (c) Experimental XRD patterns of as-grown Zn/Al(NO_3^-)-LDH films synthetized on Al foils for growth times 2 h, 15 h, 24 h. (d) Calculated XRD patterns for Zn/Al(NO_3^-)-LDH (space group *R-3m*, *a* = *b* = 3.081 Å, *c* = 22.778 Å, COD No. 3000048) and Al (space group *Fm-3m*, *a* = *b* = *c* = 4.046 Å, COD No. 9011602). Red and blue vertical solid lines mark the main XRD lines for a visual comparison among experimental and calculated patterns. XRD patterns of LDHs are indexed according to the upper labels. Two diffraction lines, Al (111) and Al (200), originate from the aluminum foil. The shoulders in the (003) and (006) basal peaks in the measured XRD patterns might be attributed to intercalation of CO_3^{2-} anions due to atmospheric contamination of CO_2 during growth and exposure to ambient air.

Table S1. Crystal lattice parameters calculated by a multi-peak fitting procedure for the three experimental XRD patterns displayed in Figure S2 (a)-(c). The basal spacing *c*' is calculated by the formula $c' = 1/2 \cdot (d_{003} + 2d_{006})$, the interlamellar spacing d_{int} is estimated by *c*' assuming a 4.8 Å thick brucite–like sheet (i.e., $d_{int} = c' - 4.8$ Å). The unit cell parameters are calculated as follows: c-axis parameter $c = 3 \cdot c'$, lattice parameter $a = 2 \cdot d_{110}$. Average crystallite sizes *D* in the *c* direction were estimated from the Scherrer equation using the full width at half maximum (FWHM) of the basal reflection plane (006): $D = 0.89\lambda/\beta\cos(\theta)$, where $\lambda = 0.15406$ nm is the wavelength of Cu K_α X-ray radiation, β is the FWHM of the diffraction peak in radians and θ is the diffraction angle.

LDH sample	Growth time (h)	d_{003} (Å)	d_{006} (Å)	*c*' (Å)	d_{int} (Å)	Lattice constants		Crystallite size in *c* direction (nm)
						c (Å)	*a* (Å)	
Zn/Al(NO_3^-)	2	8.77	4.43	8.82	4.0	26.45	3.06	13.11
Zn/Al(NO_3^-)	15	8.76	4.43	8.81	4.0	26.43	3.06	15.00
Zn/Al(NO_3^-)	24	8.69	4.43	8.78	4.0	26.32	3.06	13.64

S3. ENERGY DISPERSIVE X-RAY DIFFRACTION (EDXD) OF Zn/Al(NO_3^-)-LDH FILMS

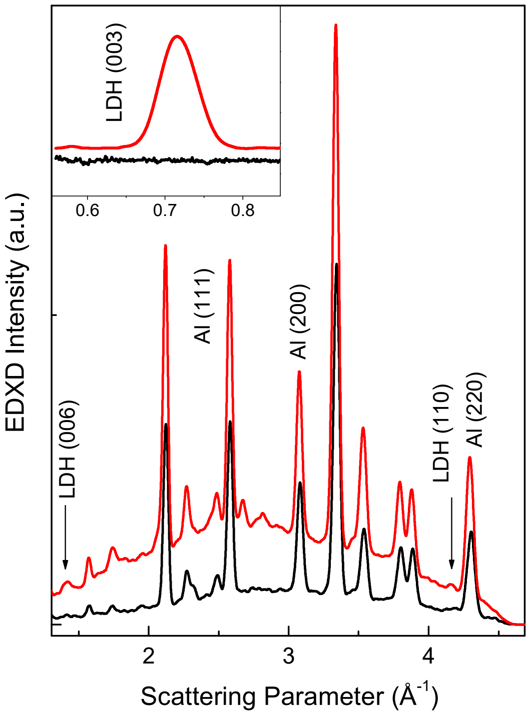

Figure S3. EDXD patterns collected upon the commercial aluminium foil (substrate, black line) and the Zn/Al(NO_3^-)-LDH film deposited on the substrate (red line). The aluminium foil substrate exhibits several spurious reflections in addition to the Al indexed ones. These substrate reflections are found in the patterns related to the deposited film, since the x-ray beam is highly penetrating (energy up to 50keV). Nevertheless, the pattern related to the Zn/Al(NO_3^-)-LDH film clearly exhibits the signatures of the basal plane (003) reflection (inset of the figure), the (006) reflection, and the (110) non-basal reflection.

S4. PL SPECTRA FROM DIFFERENT Zn/Al(NO_3^-)-LDH SAMPLES

The emerging UV PL features upon dehydration in vacuum were reproducibly measured from all the fabricated LDH samples, as well as the broad visible emission band in ambient conditions (i.e., room temperature, ambient moisture, hydrated samples).

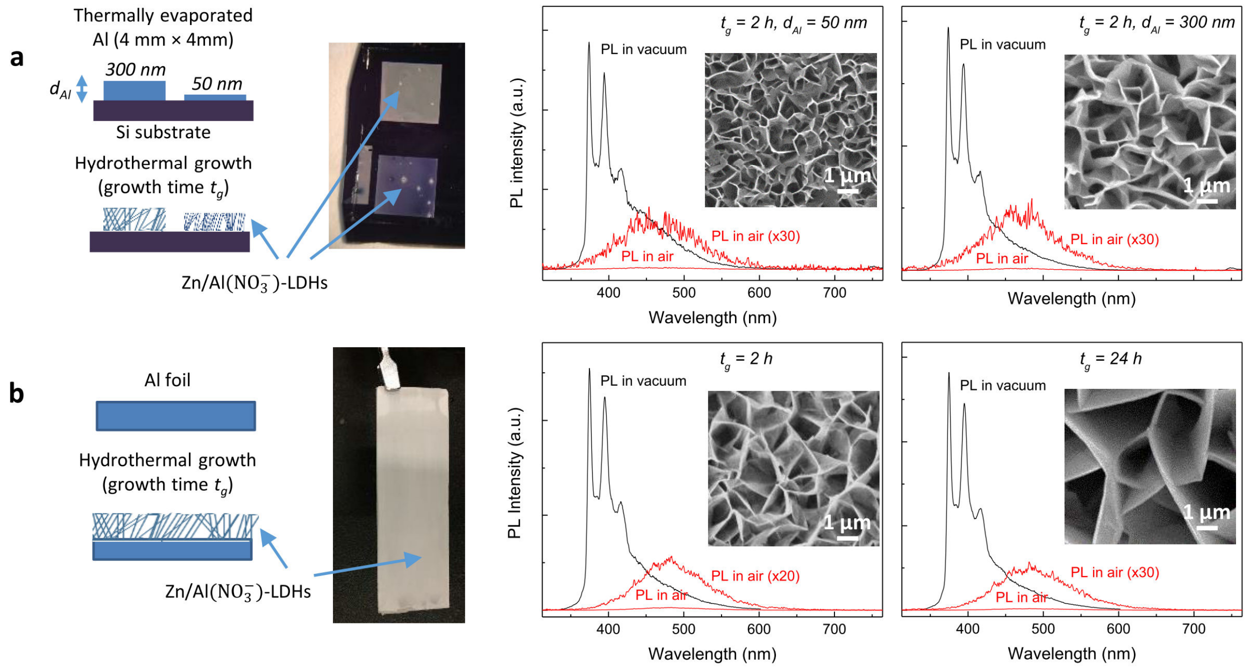

Figure S4. Schematic representation of the fabrication procedure, optical photo, room temperature PL spectra in air (red lines) and in vacuum (black lines), and scanning electron microscope (SEM) images (insets) of Zn/Al(NO_3^-)-LDHs grown (a) on Al patterned Si substrate with 4 mm × 4 mm thermally evaporated Al pads, 300 nm and 50 nm tick (d_{Al}), growth time 2 h (t_g), and (b) on Al foil, t_g = 2 h and 24 h. The same switchable PL behavior was found for all the evaluated samples.

S5. NUMERICAL SIMULATION OF THE SOLUTION CHEMICAL SPECIATION

Speciation plots to evaluate chemical species present in the nutrient solution were obtained from HySS, 4.0.31, Hyperquad Simulation and Speciation software[2]. Equilibrium constants are calculated at 80 °C by estimating conditional equilibrium constants. Details concerning the calculations are reported in a previous study[3]. In particular, the formation constant of aluminum hydrolysis products are taken from ref.[4]. Chemical reactions relevant to the formation of Zn/Al-LDH on aluminum substrates in the aqueous solution are discussed in refs.[5-7].

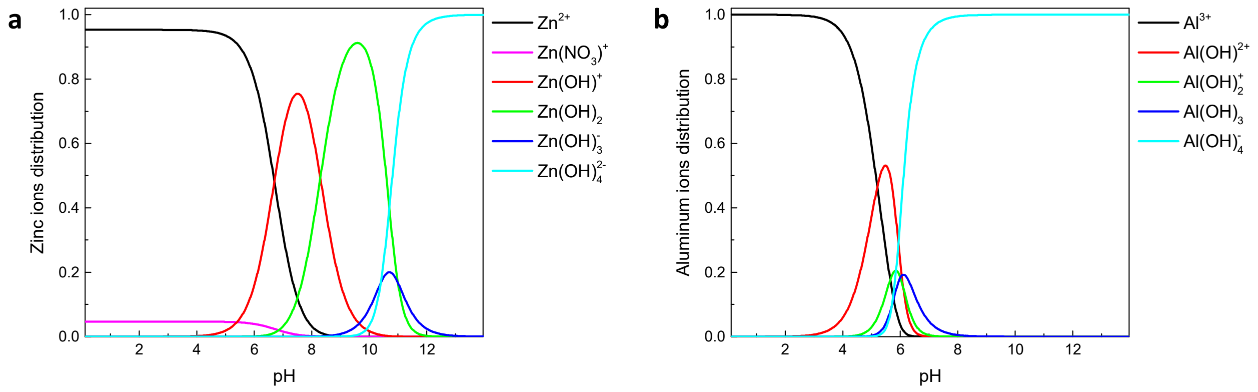

Figure S5. pH–dependent speciation plots (80 °C) showing the relative concentration of (a) the zinc complex species and (b) the aluminum complex species, as a function of pH for solutions containing 10 mM zinc nitrate hexahydrate [$Zn(NO_3)_2·6H_2O$] and 10 mM hexamethylenetetramine (HMT, $C_6H_{12}N_4$).

$Zn/Al(NO_3^-)$-LDH can be formed by the $Zn(OH)_4^{2-}$, $Al(OH)_4^-$, and nitrate NO_3^- ions according to the reaction

$$Al(OH)_4^- + Zn(OH)_4^{2-} + 2\ NO_3^- + H_2O \leftrightarrows Zn/Al(NO_3^-)\text{-LDH} \qquad (1)$$

NO_3^- anions are almost all free in the solution (see Figure S4(a)) and can be hosted in the LDH lattice due to electrostatic interactions. Al complex $Al(OH)_4^-$ is provided by the Al substrate as follows:

$$2Al + 2OH^- + 6H_2O \leftrightarrows 2Al(OH)_4^- + 3H_2 \qquad (2)$$

As pointed out by Guo at al.[8], when Al substrates are placed in solutions of high pH, the surface is partially dissolved generating surface aluminum oxides. They also report that formation of alumina may occur at lower pH (6.5) in presence of Zn^{2+} ions. In the basic solution, $Al(OH)_4^-$ is provided by Al_2O_3 by the hydroxide reaction

$$Al_2O_3 + 3H_2O + 2OH^-(aq) \leftrightarrows 2Al(OH)_4^- \qquad (3)$$

These observations are confirmed by the speciation plot for aluminum species reported in Figure S4(b), in which $Al(OH)_4^-$ species forms already at pHs around 6.

As for the Zn complex $Zn(OH)_4^{2-}$, it is provided by the zinc nitrate hexahydrate by the hydrothermal reaction

$$Zn(NO_3)_2·6H_2O \leftrightarrows Zn^{2+} + 2NO_3^- + 6H_2O \qquad (4)$$

followed by the hydroxide reaction

$$Zn^{2+} + 4OH^- \leftrightarrows Zn(OH)_4^{2-} \qquad (5)$$

It is worth to point out that, as noted by Cho et al.[7], the $Zn(OH)_4^{2-}$ complex may competitively react with $Al(OH)_4^-$ and NO_3^- to form $Zn/Al(NO_3^-)$-LDH (reaction 1) and with OH^- to form ZnO [$Zn(OH)_4^{2-} \leftrightarrows ZnO + H_2O + 2OH^-$]. However, as long as Al complex is available in nutrient solution, $Zn(OH)_4^{2-}$ reacts preferentially with $Al(OH)_4^-$ to form $Zn/Al(NO_3^-)$-LDH, whose reaction it has been shown to be thermodynamically favored. This is the case of LDHs grown on a thick Al substrate (e.g., an aluminum foil), as those used in the present paper.

S6. PL SPECTRA FROM HMT AND ZINC NITRATE HEXAYDRATE

PL spectra from samples of HMT and zinc nitrate hexahydrate salts used as ingredients in the aqueous LDH growth solution were recorded both in air and in vacuum at the same irradiation conditions of the LDH samples. No detectable PL was measured from bare Al foils.

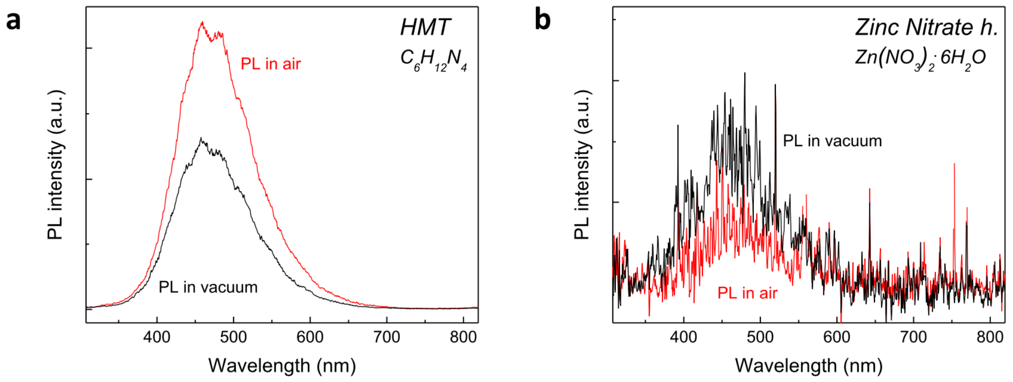

Figure S6. Room temperature PL spectra measured in air and in vacuum from samples of (a) HMT ($C_6H_{12}N_4$), and (b) zinc nitrate hexahydrate [$Zn(NO_3)_2 \cdot 6H_2O$] salts. Interestingly, an intense broad visible PL band was measured both in air and in vacuum from the HMT sample. However, no additional PL features emerged when the PL was measured in vacuum condition.

S7. NON-NORMALIZED PL SPECTRA AS A FUNCTION OF TEMPERATURE

Figure 4(a) of the manuscript displays normalized PL of Zn/Al(NO_3^-)-LDH nanosheets as a function of temperature. Normalization was aimed to improve the graphical visualization of the effects of temperature variation on PL spectral lineshape (see colour plot in Figure 4(a)). Non-normalized PL spectra vs. temperature (40 – 300 K) show an almost temperature independent behavior below 250 K and PL integrated intensity slightly increases towards 300 K. Along with this general trend, some fluctuations and discontinuities may be observed at temperatures lower than 250 K.

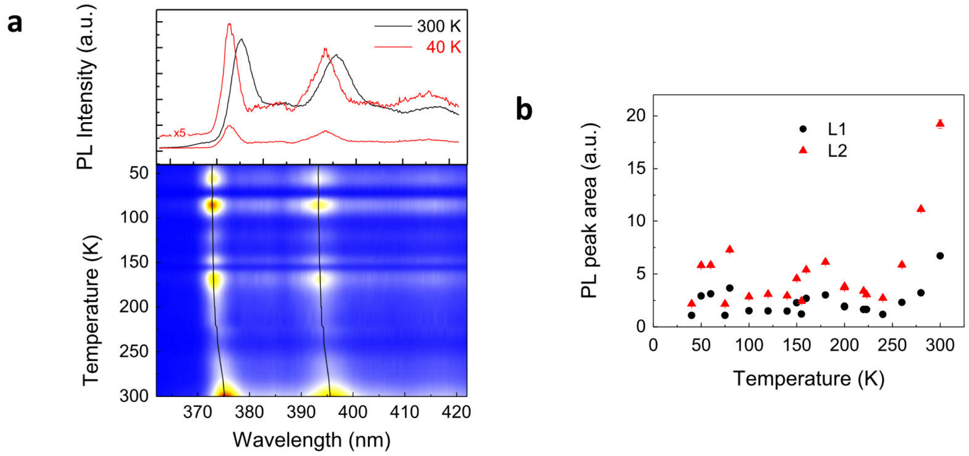

Figure S7. Non-normalized PL of Zn/Al(NO_3^-)-LDH film synthetized on Al foil (2 h growth time) as a function of temperature. (a) PL spectra at 300 K and 40 K (upper panel) and colour plot of the PL spectra measured from 40 K to 300 K (lower panel). Solid lines in the PL map are the peak positions of L1 and L2 lines extracted by the least-squares fitted measured spectra. (b) L1 and L2 peak areas versus temperature.

S8. MORE RESULTS OF DEPENDENCE OF PL FEATURES ON LASER FLUENCE

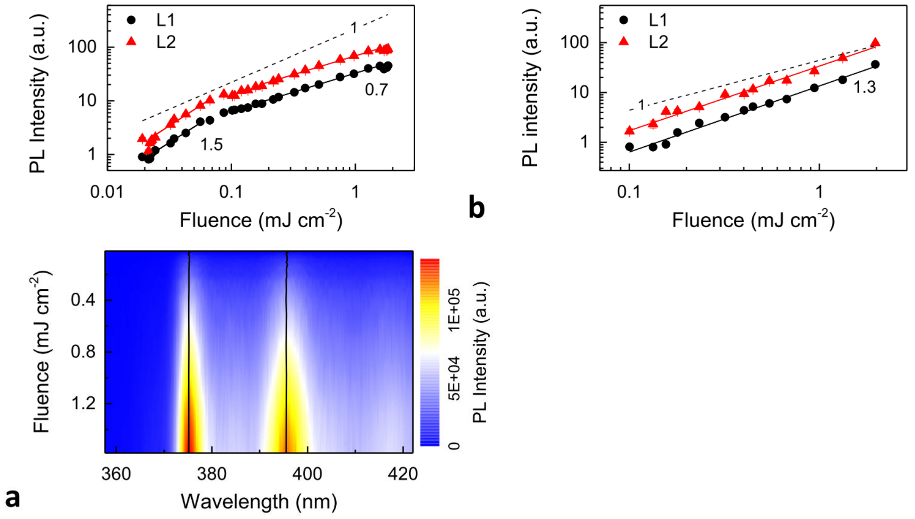

Figure S8. (a) Room temperature integrated PL intensity of L1 and L2 peaks (the solid lines are fits to a power law) and colour plot of the measured PL spectra as a function of the laser excitation fluence. Solid lines in the PL map are the peak positions of the L1 and L2 lines extracted by the least-squares fitted measured spectra. (b) Integrated PL intensity of L1 and L2 peaks vs. laser fluence reported in the manuscript (see Figure 4(d)), shown here for comparison purpose.

S9. TEMPERATURE- DEPENDENT TRPL OF L2 PL FEATURE

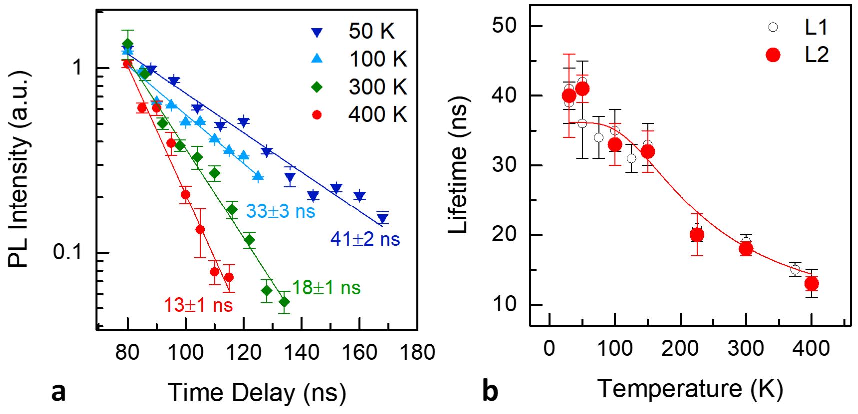

Figure S9. (a) Semilogarithmic decay traces of the integrated PL intensities relevant to the L2 feature measured at 50, 100, 300, and 400 K. The solid lines are the best fit curves of the experimental data by a monoexponential decay function. PL integrated intensities were scaled to 1 for a better visual comparison. (b) Lifetime data calculated for the L2 PL feature (closed symbols) superimposed to the TRPL data (open symbols) and best fit curve (solid line) of L1 taken from Figure 5(d) of the main text.

S10. SUMMARY OF LITERATURE DATA FOR LDH BAND STRUCTURE RECONSTRUCTION

Many works report on density functional theory (DFT) calculations of electronic band structure, total densities of states (TDOS), and partial densities of states (PDOS) of $M^{2+}/M^{3+}(A^{n-})$-LDHs (M^{2+} = Mg^{2+}, Mn^{2+}, Fe^{2+}, Co^{2+}, Ni^{2+}, Cu^{2+}, Zn^{2+}; M^{3+} = Al^{3+}, Ga^{3+}, Cr^{3+}, Fe^{3+}; A^{n-} = Cl^-, CO_3^{2-}, NO_3^-, etc.). Bandgap energy is calculated as the difference between conduction band minimum (CBM) and valence band maximum (VBM), or, according to a molecular cluster modelling approach, between lowest unoccupied molecular orbital (LUMO) and highest occupied molecular orbital (HOMO) levels. Orbital contribution of individual atomic species to VBM (HOMO) and CBM (LUMO) are also reported. Besides DFT results, experimental bandgap values are available as well. Interestingly, as a general result all reported LDH compounds behave as a direct bandgap semiconductor. Table S2 summarizes some literature data relevant to band structure reconstruction of $M^{2+}/M^{3+}(A^{n-})$-LDHs, and in particular to that of $Zn/Al(A^{n-})$-LDHs. Although a dedicated DFT study will be mandatory, in the manuscript we took advantage of these literature data to discuss the unprecedented PL of LDH nanosheet samples.

Table S2. Summary of some literature data from DFT calculations and experimental results for band structure reconstruction of Zn^{2+} or Al^{3+} based $M^{2+}/M^{3+}(A^{n-})$-LDHs. Bandgap energy, orbital contributions to VBM (HOMO) and CBM (LUMO), and the number of the corresponding Supplementary Reference are indicated.

LDH sample					
$M^{2+}/M^{3+}(A^{n-})$	*molar ratio M^{2+}/M^{3+}*	*bandgap energy (eV)*1*	*VBM (HOMO)*	*CBM (LUMO)*	*Ref.*
$Zn/Al(CO_3^{2-})$	3:1	$(5.7)^{EXP.}$	O-2p	Zn-4s, Al-3s	9
$Zn/Ga(CO_3^{2-})$	3:1	$(5.6)^{EXP.}$	O-2p	Zn-4s, Ga-4s	9
$Zn/Al(NO_3^-)$	6:1	$(5, 3.85, 3.25)^{EXP.}$	O-2p	Zn-4s, Al-3s	10
$Zn/Al(Cl^-)$	2:1	$3.49, (3.22)^{EXP.}$	O-2p, Cl-2p	Zn-4s	11
$Zn/Al(NO_3^-)$	2:1	$(5.24, 3.83)^{EXP.}$	O-2p	Zn-4s, Al-3s	12
$Zn/Al(NO_3^-)$	2:1	$(4.8, 3.75)^{EXP.}$	O-2p	Zn-4s, Al-3s	13
$Zn/Al(Cl^-)$	2:1	3.01	O-2p	Zn-4s	14
$Zn/Al(CO_3^{2-})$	2:1	$(3.54)^{EXP.}$	Zn-3d, O-2p	Zn-4s	15
$Zn/Ti(Cl^-)$	2:1	3.00	O-2p	Zn-4s	16
$Zn/Cr(Cl^-)$	2:1	2.63	O-2p	Cu-3d	16
$Mg/Al(Cl^-)$	2:1	$4.63\ (4.18)^{EXP.}$	O-2p, Cl-2p	H-1s, Mg-2p	11
$Ni/Al(Cl^-)$	2:1	2.33	O-2p, Cl-2p, Ni-3d	O-2p, Ni-3d	11
$Mg/Al(Cl)^-$	2:1	3.93	O-2p	H-1s, Mg-2p	14

*[1] The superscript $^{EXP.}$ indicates bandgap energy values calculated by experimental methods (e.g., UV-vis absorption, UV-vis diffuse reflectance spectra).

S11. UV-VIS ABSORPTION SPECTRA AND OPTICAL BANDGAP ESTIMATION

UV-VIS absorbance spectra of $Zn/Al(NO_3^-)$-LDH films grown in-situ on aluminium foils (growth times 2h, 15 h, 24 h) were measured with a Perkin Elmer Lambda 9 spectrophotometer, and are displayed in Figure S10 (a). Absorbance measurements were performed on as-grown samples in

ambient condition (i.e., humid in air, room temperature). An estimation of the optical bandgap width was obtained by means of the commonly used Tauc's equation,[17,18] given as $(\alpha h\nu)^{1/n} = B(h\nu - Eg)$, where $h\nu$ is photon energy, α is the absorption coefficient, Eg is the optical bandgap, B is a proportional constant, and n = ½ for allowed direct transition mode. Tauc's plot in Figure S10(b) reports $(\alpha h\nu)^2$ estimated by[19] $(Ah\nu)^2$, where A is the absorbance, as a function of photon energy. Bandgaps were calculated by means of the intercepts to the $(Ah\nu)^2$=0 axis of the extrapolated linear region of the Tauc's plot. Three optical bandgaps, namely E_{g1}, E_{g2}, E_{g3}, were estimated for the measured LDH samples and results are summarized in Table S3.

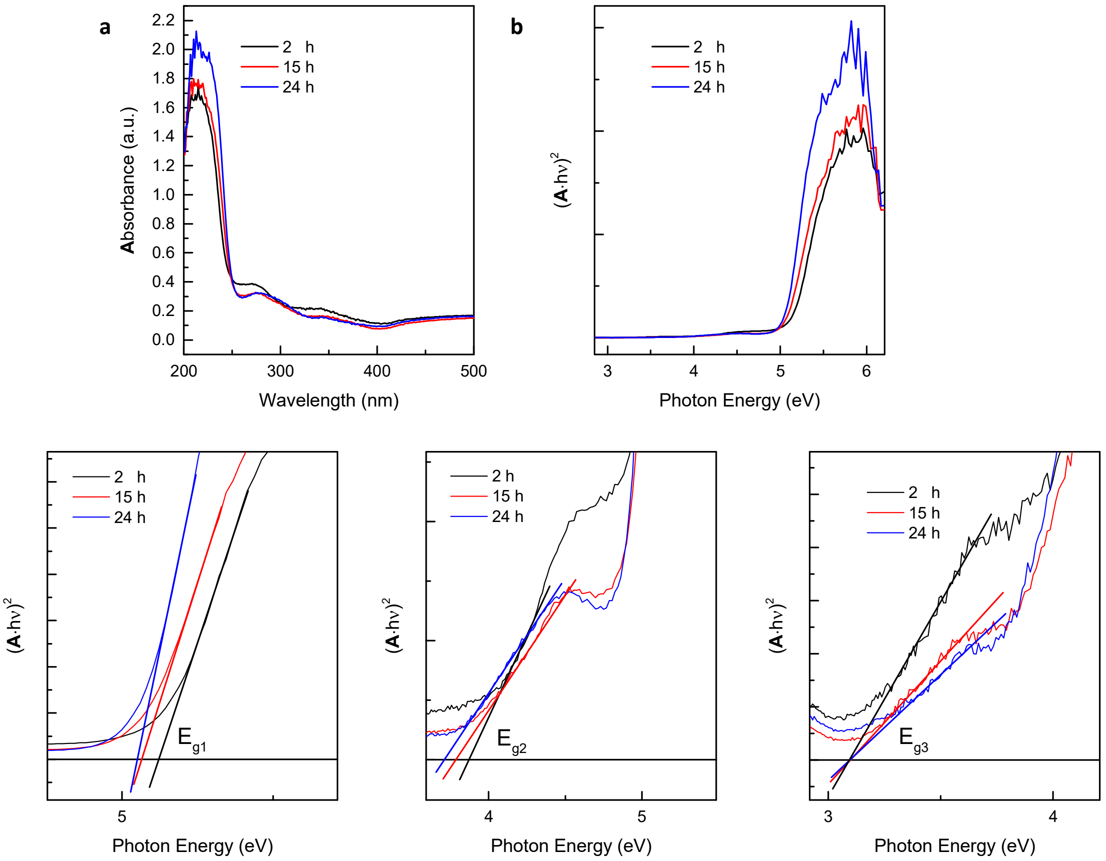

Figure S10. (a) UV-vis absorbance spectra, measured in air and room temperature, and (b) Tauc's plot of as-grown Zn/Al(NO_3^-)-LDH films synthetized on Al foils for growth times 2 h, 15 h, 24 h. (c) Zoomed views of the Tauc's plot at different photon energy regions for estimation of optical band gaps E_{g1}, E_{g2}, E_{g3}.

Table S3. Summary of optical bandgaps E_{g1}, E_{g2}, E_{g3} calculated from Tauc's plots displayed in Figure S10(c) for Zn/Al(NO_3^-)-LDH films prepared on Al foils (growth times 2 h, 15 h, 24 h).

LDH sample				
$M^{2+}/M^{3+}(A^{n-})$	*Growth time (h)*	*Band gap energy E_{g1} (eV) (±0.05 eV)*	*Band gap energy E_{g2} (eV) (±0.05 eV)*	*Band gap energy E_{g3} (eV) (±0.05 eV)*
Zn/Al(NO_3^-)	2	5.12	3.89	3.15
Zn/Al(NO_3^-)	15	5.07	3.78	3.15
Zn/Al(NO_3^-)	24	5.05	3.70	3.15

SUPPLEMENTARY REFERENCES

[1] Momma, K. & Izumi, F. VESTA 3 for three-dimensional visualization of crystal, volumetric and morphology data *J. Appl. Crystallogr.* **44** 1272–1276 (2011).

[2] Alderighi, L., Gans, P., Ienco, A., Peters, D., Sabatini, A. & Vacca, A. Hyperquad simulation and speciation (HySS): a utility program for the investigation of equilibria involving soluble and partially soluble species *Coordin. Chem. Rev.* **184** 311–318 (1999).

[3] Arrabito, G., Errico, V., Zhang, Z., Han, W. & Falconi, C. Nanotransducers on printed circuit boards by rational design of high-density, long, thin and untapered ZnO nanowires *Nano Energy* **46** 54–62 (2018).

[4] Oehman, L. O. Equilibrium and structural studies of silicon(IV) and aluminum(III) in aqueous solution. 17. Stable and metastable complexes in the system hydrogen (+)-aluminum(3+)-citric acid *Inorg. Chem.* **27** 2565–2570 (1988).

[5] Liu, J., Huang, X., Li, Y., Sulieman, K. M., He, X. & Sun, F. Facile and Large-Scale Production of ZnO/Zn/Al Layered Double Hydroxide Hierarchical Heterostructures *J. Phys. Chem. B* **110** 21865–21872 (2016).

[6] Baek, S. H., Naam, G. H. & Park, I. K. Morphology controlled growth of ZnAl-layered double hydroxide and ZnO nanorod hybrid nanostructures by solution method *RSC Adv.* **5** 59823–59829 (2015).

[7] Cho, S., Jung, S. H., Jang, J. W., Oh, E. & Lee, K. H. Simultaneous Synthesis of Al-Doped ZnO Nanoneedles and Zinc Aluminum Hydroxides through Use of a Seed Layer *Cryst. Growth Des.* **8** 4553–4558 (2008).

[8] Guo, X., Xu, S., Zhao, L., Lu, W., Zhang, F., Evans, D. G. & Duan, X. One-Step Hydrothermal Crystallization of a Layered Double Hydroxide/Alumina Bilayer Film on Aluminum and Its Corrosion Resistance Properties *Langmuir* **25** 9894–9897 (2009).

[9] Ahmed, N., Shibata, Y., Taniguchi, T. & Izumi, Y. Photocatalytic conversion of carbon dioxide into methanol using zinc–copper–M(III) (M=aluminum, gallium) layered double hydroxides *J. Catal.* **279** 123–135 (2011).

[10] Salih, E. Y., Sabri, M. F. M., Sulaiman, K., Hussein, M. Z., Said, S. M., Usop, R., Salleh, M. F. M. & Bashir, M. B. A. Thermal, Structural, Textural and Optical Properties of ZnO/$ZnAl_2O_4$ Mixed Metal Oxide-Based Zn/Al Layered Double Hydroxide *Mater. Res. Express* **5** 116202 (2018).

[11] Xu, S. M., Pan, T., Dou, Y. B., Yan, H., Zhang, S. T., Ning, F. Y., Shi, W. Y. & Wei, M. Theoretical and Experimental Study on $M^{II}M^{III}$-Layered Double Hydroxides as Efficient Photocatalysts toward Oxygen Evolution from water *J. Phys. Chem. C* **119** 18823–18834 (2015).

[12] Ahmed, A. A. A., Talib, Z. A., Hussein, M. Z. & Zakaria, A. Zn–Al layered double hydroxide prepared at different molar ratios: Preparation, characterization, optical and dielectric properties *J. Solid State Chem.* **191** 271–278 (2012).

[13] Babakhani, S., Talib, Z. A., Hussein, M. Z. & Ahmed, A. A. A. Optical and Thermal Properties of Zn/Al-Layered Double Hydroxide Nanocomposite Intercalated with Sodium Dodecyl Sulfate *J. Spectrosc.* **2014** 467064 (2014).

[14] Wang, G., Rao, D., Li, K. & Lin, Y. UV Blocking by Mg−Zn−Al Layered Double Hydroxides for the Protection of Asphalt Road Surfaces *Ind. Eng. Chem. Res.* **53** 4165–4172 (2014).

[15] Zhao, Y., Chen, G., Bian, T., Zhou, C., Waterhouse, G. I. N., Wu, L. Z., Tung, C. H., Smith, L. J., O'Hare, D. & Zhang, T. Defect-Rich Ultrathin ZnAl-Layered Double Hydroxide Nanosheets for Efficient Photoreduction of CO_2 to CO with Water *Adv. Mater.* **27** 7824–7831 (2015).

[16] Xu, S. M., Yan, H. & Wei, M. Band Structure Engineering of Transition-Metal-Based Layered Double Hydroxides toward Photocatalytic Oxygen Evolution from Water: A Theoretical–Experimental Combination Study *J. Phys. Chem. C* **121** 2683–2695 (2017).

[17] Tauc, J., Grigorovici, R. & Vancu, A. Optical Properties and Electronic Structure of Amorphous Germanium *Phys. Stat. Sol. B.* **15** 627 (1966).

[18] Davis, E. A., Mott, N. F Conduction in non-crystalline systems V. Conductivity, optical absorption and photoconductivity in amorphous semiconductors *Phil. Mag. A.* **22** 903–922 (1970).

[19] Hernández-Pagán, E., O'Hara, A., Arrowood, S., McBride, J., Rhodes, J., Pantelides, S. & Macdonald, J., Transformation of the Anion Sublattice in the Cation-Exchange Synthesis of Au_2S from $Cu_{2-x}S$ Nanocrystals *Chem. Mater.* **30** 8843 – 8851 (2018).
